# A revised trapped melt model for iron meteorites applied to the IIIAB group

**DOI:** 10.1111/maps.13740

**Published:** 2021-10-18

**Authors:** Nancy L. Chabot, Bidong Zhang

**Affiliations:** ^1^ 70603 Johns Hopkins University Applied Physics Laboratory 11100 Johns Hopkins Rd Laurel Maryland 20723 USA; ^2^ Department of Earth, Planetary, and Space Sciences University of California, Los Angeles Los Angeles California 90095‐1567 USA

## Abstract

As the largest magmatic iron meteorite group, the IIIAB group is often used to investigate the process of core crystallization in asteroid‐sized bodies. However, previous IIIAB crystallization models have not succeeded in both explaining the scatter among IIIAB irons around the main crystallization trends and using elemental partitioning behavior consistent with experimental determinations. This study outlines a revised approach for modeling the crystallization of irons that uses experimentally determined partition coefficients and can reproduce the IIIAB trends and their associated scatter for 12 siderophile elements simultaneously. A key advancement of this revised trapped melt model is the inclusion of an effect on the resulting solid metal composition due to the formation of troilite. The revised trapped melt model supports the previous conclusion that trapped melt played an important role in the genesis of IIIAB irons and matches the trace element fractionation trends observed in the Cape York suite as due to different amounts of trapped melt. Applying the revised trapped melt model to 16 elements as well as S and Fe, the bulk composition of the IIIAB core is found to have a composition consistent with that expected from a chondritic precursor for refractory siderophile elements but with evidence for depletions of more volatile elements. The bulk S composition of the IIIAB core is estimated as 9 ± 1 wt%, implying that a substantial amount of S‐rich material from the IIIAB core is underrepresented in our meteorite collections. Future applications of the revised trapped melt model to other magmatic iron meteorite groups can enable comparisons between the core compositions and crystallization processes across the early solar system.

## Introduction

The IIIAB group is the largest magmatic iron meteorite group, with over 300 members, making it well suited to investigate the process of core crystallization of asteroid‐sized planetesimals. Previous studies have described strong evidence that the IIIAB core solidified by fractional crystallization (e.g., Scott 1972; Jones and Drake 1983; Haack and Scott 1993; Ulff‐Møller 1998; Chabot 2004) but that the manner of crystallization also trapped pockets of metallic melt during that process (Wasson 1999, 2016; Wasson and Choi 2003). In particular, the IIIAB Cape York meteorites appear to define a mixing trend with different amounts of trapped melt, as evidenced by the troilite nodules that formed from that trapped melt, during one snapshot of crystallization of the IIIAB core (Esbensen and Buchwald 1982; Esbensen et al. 1982). Given these observations, Wasson (1999) introduced a model for the IIIAB elemental trends, with IIIAB irons falling between the solid metal and liquid metal tracks from fractional crystallization of the core, as a result of different amounts of trapped melt during the formation of each individual iron meteorite specimen. This trapped melt model provides a compelling conceptual model to explain the inherent spread in IIIAB irons about the fractional crystallization trend.

**Fig. 1:**
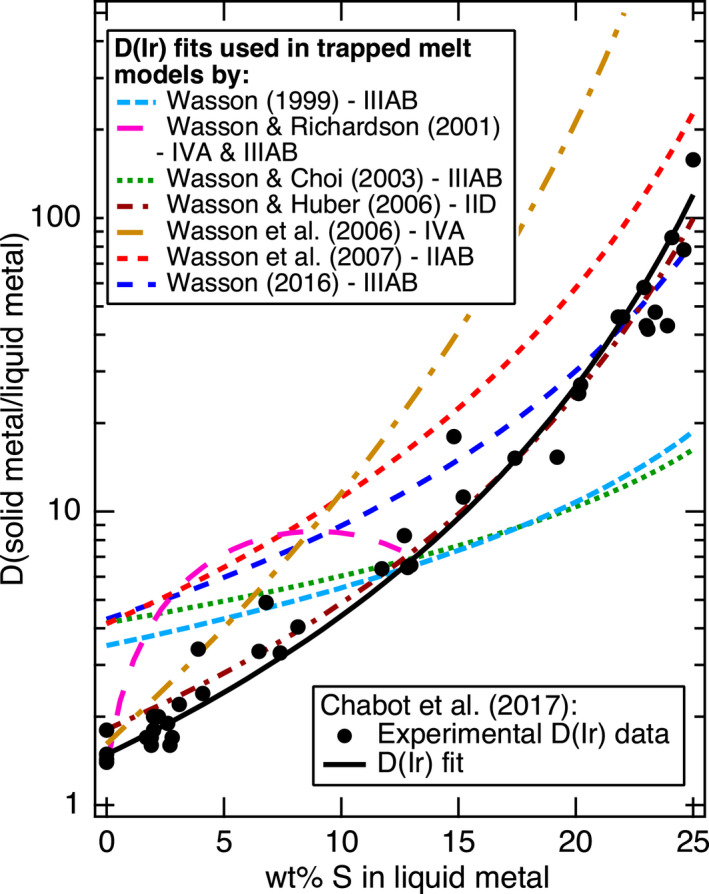
Parameterizations of D(Ir) used in previous trapped melt models have differed from experimental determinations of D(Ir) and from study to study. Experimental data are from Willis and Goldstein (1982), Jones and Drake (1983, 1986), Jones and Malvin (1990), Fleet et al. (1999), Chabot et al. (2003, 2007, 2017)

However, a major issue with the trapped melt model calculations to date is that they have used inconsistent partitioning behaviors between the different studies that differ from experimentally determined values. This is illustrated in Fig. 1, which shows that the values of the solid metal/liquid metal partition coefficient (*D*) of Ir used in the IIIAB models (Wasson 1999, 2016; Wasson and Richardson 2001; Wasson and Choi 2003) differ considerably from the experimental data. A consistent set of experimentally determined *D*(Ir) values have been produced in multiple different labs in different studies over multiple decades, as shown on Fig. 1 (Willis and Goldstein 1982; Jones and Drake 1983, 1986; Jones and Malvin 1990; Fleet et al. 1999; Chabot et al. 2003, 2007, 2017). Also, other modeling studies since the original model of Wasson (1999) have applied the trapped melt model to other magmatic iron meteorite groups (Wasson and Richardson 2001; Wasson and Huber 2006; Wasson et al. 2006, 2007), and different values of *D*(Ir) have been used to model different iron meteorite groups, as also shown in Fig. 1. In any model, the expression of *D*(Ir) is just a means to mathematically represent the partitioning behavior; however, having all models use the same *D*(Ir) expression and having that expression be consistent with the experimental determinations of *D*(Ir) would be more physically plausible. The trapped melt models listed in the legend of Fig. 1 have also modeled Ga, Ge, As, W, and Au, and parameterizations of these elements have also differed from the experimental data but to a lesser degree than for Ir.

In contrast, Chabot (2004) used a parameterization for *D*(Ir) derived from experimental data to model the IIIAB group by fractional crystallization. The model of Chabot (2004) was able to generally match the overall IIIAB elemental trends but provided no explanation for the spread in IIIAB irons about that trend. In particular, mixing between the solid metal and liquid metal tracks in the model of Chabot (2004) did not match the IIIAB iron meteorite data.

In this work, we present a revised trapped melt model that adopts the concept of trapped melt as suggested by Wasson (1999) but utilizes elemental partitioning parameterizations derived from laboratory experiments (Chabot et al. 2017). Previously, trapped melt models have not included any effects on the element chemistry due to the formation of troilite and have mathematically just used mixtures between solid metal and liquid metal to model trapped melt. The revision to the trapped melt model is to include an effect on the element chemistry due to the formation of troilite from the trapped melt. Here, we introduce the revised trapped melt model, and then, we apply it to the IIIAB group.

## The Revised Trapped Melt Model

The fundamental basis of the fractional crystallization model employed in this work is a straightforward loop with equations derived from the principles of mass balance. At the start of the model, the metallic core is defined to have an initial composition and to be completely liquid. Crystallization then proceeds, producing solid metal and resulting in a slightly changed remaining metallic liquid composition. At the first crystallization step, the composition of the solid metal and liquid metal is calculated as:

(1) $$C_{S}\left(E\right)=\frac{\left(C_{o}\left(E\right)\times D\left(E\right)\right)}{\left(1-f+f\times D\left(E\right)\right)}$$

(2) $$C_{L}\left(E\right)=\frac{C_{S}\left(E\right)}{D\left(E\right)}$$

where *C*_o_(*E*) is the original weight concentration of the element *E* in the initial completely molten core, *C*_S_(*E*) the weight concentration of *E* in the crystallized solid metal, and *C*_L_(*E*) the weight concentration of *E* in the liquid metal following crystallization. The variable *D*(*E*) is the solid metal–liquid metal weight ratio partition coefficient for the element *E*. The quantity *f* refers to the fraction of the molten core which solidifies and was set at an initial step size of 0.001; smaller values of *f* were also explored and produced results indistinguishable from using an initial value of 0.001.

Following a crystallization step, the solid metal is envisioned as being removed from the crystallizing system, such as batch removal during crystallization. The remaining metallic liquid continues to crystallize. This is modeled by simply taking the composition of the remaining metallic liquid, *C*_L_(*E*), and using it as the original liquid composition, *C*_o_(*E*), for the next crystallization step. Equations (1) and (2) are then repeated. This loop continues until all of the metallic liquid has crystallized or the Fe‐Ni‐S cotectic composition is reached. At each crystallization step, the fraction of total solid crystallized and the fraction of the core remaining liquid are also tracked. During the model, the crystallization step size, *f*, is also adjusted to result in equal mass steps even as the fraction of the core that is liquid decreases; this is calculated as *f*_o_, the original step size (which for this study was 0.001), divided by the fraction of total liquid remaining. Comparison to a model run with a constant step size *f* of 0.001 showed no difference in the results when *f* was adjusted to provide equal mass steps. However, producing output results that are regularly spaced as a function of the percent crystallization of the core is convenient for plotting and interpreting the results.

During this fractional crystallization process, the changing liquid metal composition during crystallization will affect the partition coefficients, and hence, it is necessary in the crystallization modeling to re‐compute the partition coefficient for each element, *D*(*E*), at each crystallization step. The need to include continuously varying partition coefficient values throughout the crystallization process necessitates the use of an approach like this rather than the Rayleigh fractional crystallization equation. By using a small step size and the batch removal crystallization approach outlined in the equations and text above, the compositions produced during fractional crystallization can be modeled while also continuously varying the *D* values. Using experimental determinations of *D* values as a function of the S and P contents of the liquid metal, Chabot et al. (2017) provide parameterizations for 25 elements following the form of:

(3) $$\ln\left(D\left(E\right)\right)=\ln\left(D_{o}\left(E\right)\right)+\beta_{i}\left(E\right)\times \ln\left(\mathrm{Fe}\;\mathrm{Domains}\right)$$

The quantity *D*_0_(*E*) is the solid metal–liquid metal partition coefficient in the light element‐free Fe‐Ni system and β*_i_* (*E*) a constant specific to the element *E* being fit and the light elements *i* (S or P in this model). Fe domains are defined as the fraction of free Fe atoms available in the liquid metal and are parameterized by Chabot et al. (2017) in the Fe‐Ni‐S‐P system using speciation of FeS and Fe_3_P based on the relevant Fe binary system phase diagrams:

(4) $$\mathrm{Fe}\;\mathrm{Domains}=\;\left(1‐2X_{S}‐4X_{P}\right)/\left(1‐X_{S}‐{3X}_{P}\right)$$

where *X*_S_ and *X*_P_ correspond to the molar fraction of S and P in the liquid metal, respectively. For a liquid metal that contains both S and P, Chabot et al. (2017) recommend the weighted average approach of Jones and Malvin (1990) to calculate β:

(5) $$\beta_{\mathrm{SP}}\left(E\right)=2X_{S}/\left(2X_{S}+4X_{P}\right)\times \beta_{S}\left(E\right)+4X_{P}/\left(2X_{S}+4X_{P}\right)\times \beta_{P}\left(E\right)$$

Chabot et al. (2017) tabulate the *D*_0_, β_S_, and β_P_ values used in this model for all elements other than S, which is essentially excluded from the crystallizing solid metal at the conditions of the experiments and at conditions applicable to the crystallization of iron meteorites. Consequently, S is heavily enriched in the remaining liquid metal, and a constant value for *D*(S) of 0.01 is used in the model calculations to reflect this low solid metal solubility.

The previous model steps given in Equations ((1), (2), (3), (4), (5)) reflect a standard simple fractional crystallization approach, such as used by Chabot (2004), but with updated parameterization of the *D* values from Chabot et al. (2017). The key revision to this revised trapped melt model is to now include an effect on the element chemistry due to the formation of troilite. Previous trapped melt models, such as listed in Fig. 1, have mathematically just used mixtures between solid metal and liquid metal, such as calculated from Equations (1) and (2), to model melt trapped during crystallization. However, any liquid metal trapped will eventually cool and form mainly solid Fe‐Ni metal and troilite. Schreibersite and other accessory phases may also form, but troilite and solid Fe‐Ni metal will be the two dominant phases that solidify from an S‐bearing liquid metal, and hence for this initial revised model, we treat the formation of these other minor phases as negligible at this point. Thus, to include an effect on the element chemistry due to the formation of troilite from the trapped liquid metal, we assume a simple system where any trapped melt will solidify into troilite and solid metal, expressed by mass balance as:

(6) $$C_{L}\left(E\right)=x\times C_{\mathrm{FeS}}\left(E\right)+\left(1‐x\right)\times C_{S\_\mathrm{Trap}}\left(E\right)$$

*C*_FeS_(*E*) and *C*_S_Trap_(*E*) are the weight concentrations of element *E* in the resulting troilite and solid metal, respectively, and *x* is the fraction of the trapped liquid melt that solidifies to troilite rather than solid metal. Given that S is nearly insoluble in the Fe‐Ni solid metal that forms (Raghavan 2004), *C*_S_Trap_(*S*) in Equation (6) can be set to zero and the quantity *x* can be calculated as:

(7) $$x=C_{L}\left(S\right)/C_{\mathrm{FeS}}\left(S\right)$$

where *C*_FeS_(*S*) is the weight concentration of S in FeS, which is ~36.5 wt% as calculated from atomic weights. Many siderophile elements have very low solubility in troilite in comparison to Fe‐Ni metal, and thus, their concentrations in the troilite, *C*_FeS_(*E*), can be approximated as zero in Equation (6), resulting in a simplified equation:

$$\mathrm{if}:\;C_{\mathrm{FeS}}\left(E\right)=0$$

(8) $$\mathrm{then}:\;C_{S\_\mathrm{Trap}}\left(E\right)=\;C_{L}\left(E\right)/\left(1‐x\right)$$

Wasson (2016) noted in relation to the iron meteorite measurements that “because our goal is to obtain metal compositions, we avoid non‐metallic inclusions.” Thus, to model the metal compositions of iron meteorites, it is necessary to consider only the portion of the trapped liquid metal that forms solid metal and not troilite. In this revised trapped melt model, we make the simplifying assumption that all the elements modeled have very low solubility into troilite in comparison to solid metal and use Equation (8) to calculate the solid metal that forms from the trapped melt. This simplification is appropriate for many siderophile elements, but chalcophile elements may partition into troilite. This is discussed in Chalcophile Elements: Cr and Cu section in more detail when the revised trapped melt model is applied to the IIIAB group. Future modeling efforts can build on this initial revision for handling trapped melt by utilizing Equation (6) along with solid metal–troilite partition coefficients, which would be a key addition for interpreting certain chalcophile elements.

**Fig. 2:**
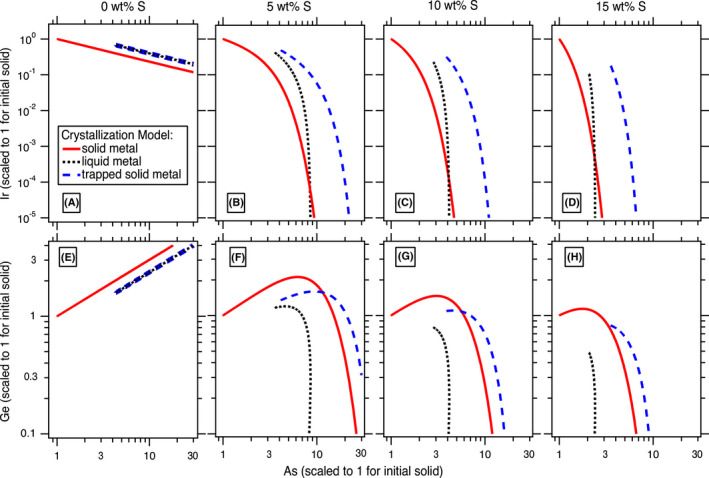
Revised trapped melt model calculations for (A–D) Ir and (E–H) Ge versus As for four different initial S concentrations. The red line represents the solid metal that forms directly by fractional crystallization from the liquid metal and is calculated from Equation (1). The black dotted line is the corresponding liquid metal during fractional crystallization and is calculated from Equation (2). The blue dashed line represents the composition that a solid metal would have that formed along with troilite when the trapped liquid metal solidified and is calculated from Equation (8)

Figure 2 provides an example of the revised trapped melt model applied to the elements Ge, As, and Ir for four different initial S concentrations. Three model calculations are shown on each graph in Fig. 2: solid metal as calculated from Equation (1), liquid metal as calculated from Equation (2), and solid metal that forms from a trapped liquid as calculated from Equation (8). In the model with 0 wt% S in Fig. 2, there is no difference between the liquid metal and the trapped solid metal trends because troilite does not form in the S‐free system and any liquid melt trapped will solidify completely to solid Fe‐Ni metal. However, in the S‐bearing models in Fig. 2, the calculated trends for the liquid metal and the trapped solid metal differ from each other as the revised model accounts for the formation of troilite on the resulting concentrations of Ge, As, and Ir in the solid metal that forms from a trapped melt. The initial S content of the model has a large effect on the modeled calculations in Fig. 2, as expected given the *D* values of the elements are highly sensitive to the S content of the liquid metal (Chabot et al. 2017).

This revised trapped melt model can be applied to plot any element trends against each other. For this work, we choose to plot the model results against As on the *x*‐axis for two main reasons. First, as noted by Wasson (1999), As and Au are preferred for plotting iron meteorite trends against because these element concentrations are determined with high relative precisions in the iron meteorite samples and have a larger range of values in a given iron meteorite group than Ni, which had been commonly used in older studies. Second, Chabot et al. (2017) discuss that *D*(Au) is more poorly understood at low S contents than other elements and hence recommend evaluating iron meteorite crystallization models by plotting against As rather than Au, and hence we following that recommendation for this study.

## IIIAB Meteorite Data

The IIIAB group is the largest magmatic iron meteorite group, and though many studies have published modeling results applied to this group as discussed in Introduction section, a complete tabulation of IIIAB iron compositions has not been previously published. Consequently, in this study, we have included Table 1, which reports elemental compositions for 257 samples that are in the IIIAB group or have been related to the IIIAB group. To avoid interlaboratory differences, we only list data generated at UCLA in Table 1 and use these data in Table 1 for our modeling study. Earlier UCLA compositional results for many of the IIIAB irons listed in Table 1 have previously been reported in Esbensen et al. (1982), Kracher et al. (1980), Malvin et al. (1984), Scott et al. (1973), Scott and Wasson (1976), Wasson (1990, 1999, 2011, 2016), Wasson et al. (1989, 1998), and Wasson and de Bon (1998) by neutron activation analysis (NAA) and atomic absorption spectrometry (for Ni in the early studies such as Scott et al. 1973; Scott and Wasson 1976; Kracher et al. 1980; Malvin et al. 1984).

**Table 1:** Elemental composition of IIIAB and related irons.

Meteorite	Cr (µg/g)	Co (mg/g)	Ni (mg/g)	Cu (µg/g)	Ga (µg/g)	Ge (µg/g)	As (µg/g)	Ru (µg/g)	Sb (ng/g)	W (µg/g)	Re (ng/g)	Os (µg/g)	Ir (µg/g)	Pt (µg/g)	Au (µg/g)
IIIAB															
Astoria^‡^	91	4.96	72.4	190	18.0	<80	3.18	13.8	<150	1.29	1744	22.0	15.8	14.9	0.492
Acuña^1^	13	5.57	100.8	137	16.6	29.0	20.7		89	0.17	<20		0.022	2.5	2.300
Aggie Creek	24	5.20	85.2	149	20.7	39.9	7.91	8.2	<150	0.68	<64		0.572	6.6	1.064
Agua Blanca^1^	33	5.17	84.8	146	21.6	44.6	8.10		61	0.62	<60		0.430	6.4	1.025
Aldama (a)^1^	65	5.12	78.5	154	20.5	44.1	5.46		55	0.76	50		0.817	8.7	0.736
al‐Ghanim (iron)^1^	106	5.10	78.4	169	19.8	41.4	5.15		43	1.00	192		2.34	11.5	0.724
Allan Hills 84165^1^	122	4.97	79.0	173	19.6	39.0	4.06		29	1.06	275		3.64	14.1	0.632
Angelica	86	4.94	73.7	176	18.3	34.9	3.57			1.24	808		9.47		0.541
Apache Junction^‡^	18	5.22	87.6	138	20.6	<50	8.85	3.6	<120	0.52	<20	<0.2	0.193	6.0	1.230
Apizaco^‡^	14	5.25	85.5	137	21.4	<60	9.35	3.7	<100	0.52	34	0.2	0.322	5.6	1.174
Apoala^1^	17	5.53	94.1	145	18.0	35.4	15.9		<100	0.21	<10		0.016	2.6	1.923
Aprel'sky	13	5.44	97.5	112	19.3	37.0	17.3		<200	0.26	<40		0.058	3.0	2.022
Ariah Park^‡^	90	4.94	77.4	167	18.4	<50	3.86		<100	1.21	878		9.14	13.4	0.573
Asarco Mexicana	13	5.20	87.0	131	20.4	44.2	8.28			0.69	30		0.271	5.2	1.085
Augusta County	16	5.06	81.9	165	19.1	36.3	5.94		<120	1.20	1030		9.55	13.4	0.782
Augustinovka^1^	59	5.53	97.0	156	18.6	37.6	19.5		148	0.24	<25		0.026	2.8	2.093
Avoca (Western Australia)	11	5.37	90.8	119	21.3	44.5	11.8		<150	0.48	44		0.404	7.4	1.378
Bagdad	14	5.02	79.1	205	19.4	39.7	4.72		<200	1.05	910		7.76	11.4	0.703
Bald Eagle	22	5.52	92.6	143	20.0	37.1	16.0		<150	0.32	<20		0.020	2.8	1.761
Balsas^1^	46	5.10	84.3	155	20.9	<60	7.24		<200	0.66	<70		0.397	6.7	0.935
Baquedano^1^	237	5.26	88.9	155	20.8	43.1	9.56		<200	0.39	<23		0.082	<2.0	1.274
Bartlett	12	5.30	86.7	122	21.0	46.0	11.3	6.6	<150	0.86	37		0.729	7.1	1.234
Bear Creek^1^	12	5.56	99.2	176	18.9	32.8	20.8		<200	0.24	<20		0.021	3.1	2.230
Bear Lodge	438	5.02	77.9	168	19.3	38.7	4.26	10.2	<150	1.07	470	4.1	5.22	13.7	0.619
Bella Roca^1^	11	5.68	100.9	133	16.7	31.1	21.4		<100	0.15	<10		0.019	1.7	2.362
Benedict	26	5.30	88.7	146	21.2	45.4	9.84		<140	0.50	<42		0.155	5.2	1.253
Billings	142	4.95	76.6	173	19.7	37.4	4.07	11.3	<150	1.17	1026	11.4	9.98	14.9	0.577
Boxhole^1^	118	4.97	76.4	172	18.7	37.2	3.89		<100	1.19	826		8.27	13.1	0.554
Brainard	95	5.33	90.6	153	22.6	45.2	11.4		91	0.42	<60		0.063	3.6	1.438
Briggsdale	24	5.23	78.1	145	20.7	<50	6.05	6.8	<120	0.93	69		0.792	8.1	0.822
Buenaventura^1^	55	5.62	97.7	123	17.5	34.5	19.2		<100	0.21	<10		0.013	1.6	2.202
Bur‐Abor^‡^	83	5.12	79.9	164	20.9	<50	6.24		<150	0.71	50		0.744	8.5	0.834
Burns^‡^	13	5.69	103.7	111	14.4	~28	23.4	1.4	155	0.14	<20	<0.14	0.022	1.9	2.588
Cabin Creek	25	5.23	84.6	210	21.5	39.6	7.40		81	0.68	47		0.765	10.2	0.999
Cacaria	64	4.97	73.1	164	18.8	35.6	3.83	13.1	<120	1.18	1079		10.6	13.6	0.588
Cape York (mean, *N* = 7)^1^ ^,^ ^2^	64	5.05	79.6	170	19.7	35.9	5.85	9.0	55	1.04	406	3.2	4.53	11.7	0.735
Camp Wood^‡^	37	4.98	73.7	155	18.4	<70	3.70	13.2	<150	1.20	1160		12.1	15.2	0.566
Campbellsville	16	5.27	85.2	138	20.4	43.8	9.25		<150	0.45	<50		0.078		1.177
Canton	108	4.98	76.8	161	19.2	35.9	3.88	10.9	<150	1.28	1072	11.7	10.0	13.6	0.585
Canyon City	128	4.96	74.0	168	18.3	36.8	3.45	12.8	<150	1.27	962		10.2	14.5	0.526
Caperr	16	5.29	87.2	151	21.7	45.3	10.8	3.6	<100	0.48	<25	<0.21	0.241	5.9	1.290
Casas Grandes^1^	98	4.99	77.8	164	19.7	37.4	4.84		<250	1.08	372		4.74	13.6	0.644
Casimiro de Abreu	19	5.22	84.8	128	21.2	41.0	7.95			0.57	28		0.314	8.0	1.024
Catalina 107^‡^	29	5.06	79.4	176	19.8	<50	5.42	10.4	<100	0.95	261	2.0	3.34	10.8	0.744
Chambord	145	4.98	72.8	173	18.8	<50	3.80	14.9	<150	1.22	1085		11.6	14.8	0.540
Chañaral^1^	36	5.12	82.0	158	21.7	43.3	6.90		<160	0.66	<40		0.224	7.8	0.933
Charcas	397	4.98	78.2	166	20.5	41.4	5.32			0.99	102		2.28	13.7	0.698
Chilkoot	93	5.08	78.4	170	20.0	39.3	4.92	9.5	<150	0.96	185		2.54	11.5	0.696
Chisenga^1^	12	5.20	88.9	120	21.0	42.0	10.7		139	0.56	39		0.518	6.5	1.232
Chulafinnee	102	4.99	74.4	173	19.3	33.7	3.98	10.8	<150	1.12	644	5.9	6.43	13.8	0.577
Chupaderos [suite]^†,^ ^1^	13	5.64	99.6	135	17.1	29.6	21.3			0.19	<40		0.020	2.5	2.272
Cleveland	14	5.34	85.3	145	20.5	41.9	11.6		<250	0.39	<20		0.085	3.5	1.382
Colton^1^	16	5.20	82.9	148	21.2	47.9	7.46		92	0.71	44		0.632	5.6	0.962
Costilla Peak^1^	160	4.96	74.6	173	18.7	33.6	3.46		<190	1.40	1030		14.0	13.4	0.506
Cumpas^1^	49	5.11	78.9	155	20.6	42.5	5.59		<100	0.88	250		3.03	9.4	0.791
Dadin^‡^ ^,^ ^§1^	22	5.18	85.6	137	21.2	<50	8.15	5.1	<150	0.59	27	0.23	0.410	7.1	1.024
Dahongliuxia^‡^	128	5.04	75.9	189	19.7	<74	4.14	10.2	<150	1.12	867	8.1	8.23	14.3	0.581
Dalton^1^	182	4.96	72.0	163	17.5	33.2	3.08		<100	1.52	1500		13.8	13.0	0.493
Davis Mountains	135	4.91	73.9	173	17.9	33.7	3.45		<200	1.29	1690		14.8	15.2	0.474
Denton County	76	5.12	80.7	163	21.1	42.7	6.75		<120	0.69	26		0.323	7.2	0.891
Dexter^1^	28	5.09	82.6	159	20.7	40.9	5.57	8.5	<150	1.04	238		2.58	12.0	0.775
Digor^‡^	54	4.98	74.8	194	18.5	<70	4.18	12.2	<150	1.38	1373	18.2	13.5	15.1	0.566
Djebel In‐Azzene^‡^	9	5.78	92.2	116	18.5	<50	24.2	1.5	<150	0.19	<30	<0.2	0.018	1.9	2.344
Dolores^‡^	106	4.98	74.8	168	18.6	<40	3.78		<100	1.23	594		7.02	14.4	0.571
Domeyko^‡^	14	5.53	94.8	112	19.2	<50	16.7	1.8	<150	0.22	<28	<0.14	0.057	3.1	1.848
Drum Mountains	21	5.13	82.3	165	20.9	41.8	6.74		<100	0.75	41		0.742	7.9	0.876
Duketon	148	5.06	75.1	164	19.8	38.1	4.21	10.5	<150	0.99	338	3.6	4.19	12.8	0.615
Dunganville^1^	17	5.03	78.4	146	20.7	38.6	6.06	8.7	68	0.96	207	1.3	2.23	12.6	0.765
Durango	32	5.09	78.7	153	20.2	40.2	5.53		<150	0.78	88		1.12	8.8	0.777
Edmore^‡^	19	5.42	89.6	121	21.1	<60	14.2	3.4	<150	0.62	<50	<0.2	0.070	4.2	1.621
El Capitan	22	5.31	86.9	150	20.8	45.1	9.07		<150	0.44	<20		0.118	4.5	1.165
El Sampal^1^	14	5.23	89.0	134	19.8	39.6	9.80	5.5	<150	0.60	40	0.35	0.610	7.9	1.280
Elyria	15	5.22	88.5	113	20.6	43.4	9.70	4.9	<120	0.54	45		0.642	6.2	1.149
Fairview^1^	85	4.98	76.6	167	19.2	37.9	4.42		64	1.09	772		7.59	11.7	0.625
Felsted^1^	12	5.12	86.8	156	20.9	44.5	7.53		41	0.71	70		0.589	7.3	1.052
Floydada^1^	15	5.35	90.9	154	20.4	41.4	12.5		105	0.33	<40		0.023	2.7	1.452
Fort Pierre	131	4.97	76.2	175	18.4	35.9	3.70	14.1	<150	1.14	791		8.51	14.0	0.544
Franceville	90	5.12	82.6	158	20.5	42.5	6.11		<100	0.69	23		0.378	6.7	0.867
Frankfort (iron)	100	5.10	79.1	163	20.3	<50	5.57	8.2	<150	0.84	98		1.75	9.9	0.753
Glasgow	117	4.96	76.7	169	18.7	38.8	4.00	10.4	<150	1.12	446	5.0	5.34	13.2	0.598
Gnowangerup	20	5.25	84.2	128	22.1	46.4	9.53		102	0.56	<85		0.347		1.253
Grant^1^	32	5.39	93.6	136	19.1	37.0	15.5		<120	0.26	<20		0.042	3.2	1.801
Grant [Breece]	34	5.41	93.6	138	20.4	37.9	16.5	2.1	142	0.34	<30	<0.3	0.044	3.1	1.885
Greenbrier County	130	4.93	73.0	177	17.8	33.3	3.26	14.7	<150	1.32	1611		14.8	15.5	0.485
Grein 005^‡^	142	4.94	73.0	169	18.2	52*	3.48	13.4	<155	1.34	1415	14.8	13.1	16.3	0.485
Grosvenor Mountains 17051^‡^,^§^	210	5.01	73.5	166	17.6	<50	3.20	13.7	<100	1.31	1812	23.3	16.5	14.0	0.471
Grosvenor Mountains 85201^1^	67	5.15	86.2	147	20.0	42.3	7.49		68	0.57	<40		0.362	6.1	1.004
Grosvenor Mountains 95522^‡^	21	5.14	79.4	159	21.2	<50	7.42		<100	0.74	43		0.650	8.9	0.871
Guilford County^1^	91	5.02	80.9	159	21.5	41.9	5.54		75	0.89	118		1.60	9.7	0.762
Guixi	107	4.96	79.0	165	20.4	39.8	4.68		95	1.13	210		2.58	11.0	0.668
Gundaring	49	5.11	85.5	171	20.6	43.9	7.31		<120	0.68	<30		0.308	7.2	0.975
Haig^1^	154	4.95	73.2	166	18.7	33.2	3.31			1.41	1576		14.7		0.480
Holliday^‡^,^§^,^1^	184	4.91	73.5	185	18.6	<50	3.39		<150	1.42	1470		13.7	15.8	0.469
Hardesty^1^	13	5.41	94.6	100	18.1	39.0	15.8		150	0.35	<30		0.088	4.6	1.805
Harriman (Om)	28	4.98	76.6	167	18.7	36.9	3.67	11.8	<120	1.19	1251	15.3	11.5	14.8	0.560
Henbury	136	4.92	74.2	155	18.3	33.4	3.22		27	1.32	1537		13.4	16.3	0.485
Hidden Valley^1^	55	5.01	80.1	167	19.7	43.4	4.70			1.16	448		4.98	9.6	0.674
High Island Creek^‡^	110	5.10	78.6	170	21.2	<50	5.42	7.0	<100	0.94	160	1.07	2.25	11.5	0.727
Hot Springs^1^	32	5.08	77.7	153	18.6	38.0	4.67		57	1.05	430		4.87	11.7	0.715
*Ider*	*104*	*4.61*	*67.8*	*153*	*19.9*	*<101*	*7.90*	*6.8*	*<150*	*0.97*	*348*	*2.6*	*2.93*	*10.0*	0.982
Ilimaes (iron)^†^,^1^	107	5.25	84.3	125	21.8	43.4	9.70		<250	0.53	<50		0.250	5.1	1.253
Iron Creek	48	4.99	79.9	158	20.1	39.6	5.69	9.7	<120	0.97	238		3.14	10.8	0.749
Itutinga	172	4.90	71.7	183	18.6	36.0	3.44		24	1.54	1497		13.5		0.521
Ivanpah	217	5.06	75.7	162	19.6	37.9	4.19		<100	1.00	466		5.41	11.6	0.639
Jianshi	54	5.18	84.5	170	21.1	44.5	8.32		150	0.70	40		0.397	5.5	1.089
Joe Wright Mountain	12	5.32	93.2	158	19.7	35.5	13.0		<100	0.32	<16		0.018	3.2	1.626
Joel's Iron	24	5.23	88.8	144	21.1	42.8	9.70		<160	0.46	<50		0.384	5.1	1.195
Juncal	11	5.08	81.5	146	21.1	40.9	6.80		<170	0.82	190		1.90	8.9	0.872
Juromenha	13	5.32	91.4	109	21.1	40.3	13.1		<100	0.40	<16		0.166	5.0	1.483
Kalkaska	142	4.91	74.6	162	17.9	33.5	3.07	14.0	<150	1.41	1434	22.1	14.5	15.3	0.496
Kayakent	15	5.09	83.4	138	20.8	44.0	6.66	7.9	<150	0.79	71		1.12	8.9	0.885
Kenton County	105	4.92	74.4	175	18.7	35.0	3.17		<100	1.59	1529		14.3	13.9	0.490
Kenton County [Williamstown]	106	4.92	73.8	177	18.6	32.6	3.13		<300	1.38	1750		15.3	15.1	0.478
Knowles	10	5.60	95.2	123	18.6	31.6	18.5		<150	0.19	<20		0.020	2.9	2.113
Kouga Mountains	60	5.61	93.5	128	19.2	35.3	17.0		<150	0.29	<20		0.021	3.1	1.859
Kyancutta	64	5.10	82.4	169	20.6	39.5	5.70		<150	0.97	135		1.72	9.3	0.804
La Porte	55	5.04	79.7	158	21.6	43.1	5.84	7.1	<150	0.90	126	0.59	1.50	10.2	0.750
Lanton	18	5.06	83.2	178	19.7	39.3	5.75		<150	0.98	398		4.50	10.2	0.808
Las Cruces^‡^	15	5.32	91.5	115	20.6	<50	12.5	3.5	<250	0.40	20	<0.12	0.177	4.4	1.459
Las Salinas	13	5.66	100.8	132	17.7	31.9	21.0		170	0.20	<30		0.024	1.7	2.256
Lenarto	49	5.30	85.9	138	20.4	43.5	8.59		<100	0.51	36		0.443	6.8	1.171
Liangcheng	64	5.10	81.9	168	21.0	45.7	6.66		59	0.76	50		0.593	9.3	0.890
Livingston (Montana)	211	4.95	75.5	178	17.7	34.9	3.74			1.45	949		11.2	14.3	0.507
Llano River^‡^ ^,^ ^1^	182	4.96	73.5	182	19.4	<55	3.66	11.6	<170	1.28	954	9.3	9.57	13.4	0.537
Longtian^‡^	153	4.96	73.2	173	19.0	<60	3.49		<150	1.32	921		9.27	13.6	0.532
Loreto	133	5.00	78.1	169	19.6	38.3	4.74		<100	1.10	340		4.27	11.7	0.686
Los Reyes	11	5.34	89.9	115	20.3	40.7	12.0		<150	0.43	<20		0.128	4.6	1.453
*Lucky Hill* ^1^	*335*	*4.28*	*58.6*	*208*	*29.4*	*<100*	*10.6*	*7.1*	*<150*	*0.94*	*33*	*<0.5*	*0.332*	*8.9*	*1.261*
Luis Lopez	15	5.26	88.8	164	21.3	41.9	9.71	3.9	<150	0.53	<30		0.155	5.0	1.187
Madoc	352	4.93	76.4	175	19.5	36.4	3.90	12.0	<150	1.19	567		6.49	13.1	0.572
Maldyak^1^	26	5.29	94.8	217	22.7	42.6	11.2		<200	0.61	45		0.491	6.3	1.393
Manitouwabing^1^	121	5.02	81.3	166	20.3	42.9	4.73		43	1.36	160		2.36	9.5	0.679
Mapleton	47	4.98	78.3	152	20.4	40.6	5.30	8.1	<150	0.89	100		1.51	10.0	0.762
Merceditas^1^	52	5.00	78.1	175	19.0	38.9	4.18		<100	1.03	314		3.72	13.6	0.632
Meteorite Hills 00400^‡^	109	5.03	75.3	158	19.4	<60	4.67		<100	1.05	247		3.11	13.2	0.664
Milly Milly	86	5.02	78.8	167	19.7	38.6	4.82	10.7	<150	1.01	257		3.10	11.6	0.641
Moorumbunna	14	5.28	85.8	121	21.5	44.0	11.1		<200	0.49	<20		0.259	5.6	1.312
Morito	110	4.94	76.5	166	18.6	35.8	3.72	14.3	<150	1.36	1090		10.5	13.7	0.522
Mount Edith	11	5.34	93.7	138	20.1	37.5	15.0		<200	0.36	<40		0.014	3.2	1.704
Mount Wegener^1^	125	4.99	75.9	158	19.3	38.1	4.27		34.0	1.01	330		3.64	11.2	0.614
Narraburra	12	5.58	101.5	127	15.6	28.7	20.1		<300	0.20	<30		0.018	1.8	2.336
Nazareth (iron)	14	5.27	91.1	128	21.0	40.3	12.4		<100	0.51	<40		0.445	6.8	1.413
New York^‡^ ^,^ ^1^	104	4.99	76.6	161	18.4	<50	3.73	10.7	<150	1.13	437	3.4	5.29	12.1	0.594
Norfolk	138	4.91	74.0	171	19.1	38.1	3.40	12.8	<150	1.42	1000	12.8	10.5	14.8	0.509
Norfork	66	5.00	78.9	170	20.2	40.1	4.73		<100	1.09	312		3.84	15.5	0.728
Norquín^1^	15	5.25	91.2	121	19.6	41.9	11.6		112	0.43	<50		0.060		1.607
Norristown	12	5.66	94.9	126	18.7	32.4	20.1		<150	0.23	<30		0.024	2.7	2.134
Northwest Africa 860^1^	37	5.22	83.6	151	20.5	<50	7.88		<100	0.63	<60		0.404	6.3	1.064
Northwest Africa 1430^1^	38	5.06	77.4	187	19.2	<60	4.55		<150	1.14	312		3.89	13.0	0.675
Northwest Africa 3208^1^	203	4.95	75.8	164	17.7	<50	3.25	17.7	<150	1.34	2174		18.9	16.5	0.478
Northwest Africa 4707^1^	35	5.18	81.8	163	22.1	<70	6.71	7.9	<150	0.71	47		0.748	7.6	0.874
Northwest Africa 4708^1^	46	5.05	78.9	189	19.3	74*	4.67		<120	1.05	289		3.88	12.5	0.672
Northwest Africa 6903^‡^	16	5.17	84.0	130	21.5	<50	8.75	3.8	<150	0.54	<21	<0.24	0.222	6.3	1.086
Northwest Africa 8370^‡^	13	5.29	84.5	116	21.4	<50	9.92	4.4	<100	0.56	25	0.30	0.553	5.9	1.195
Northwest Africa 8442^‡^	62	5.02	79.8	168	20.4	<54	5.29	8.8	<150	0.92	226	1.6	2.82	11.6	0.707
Northwest Africa 11289^‡^	18	5.56	88.7	120	20.6	<100	14.3	3.6	<150	0.33	<20	<0.3	0.084	3.8	1.682
Nossa Senhora do Livramento^‡^	44	5.00	74.9	202	19.5	<70	4.56	12.7	<150	1.24	1403	16.2	12.5	14.0	0.647
Nova Petropolis	63	5.04	75.2	158	19.9	36.5	4.22		42	1.20	1222		11.6		0.584
Nuleri	183	4.92	74.0	150	17.7	36.7	3.42	13.1	<100	1.16	1018		11.0	15.1	0.514
Nyaung^1^	113	4.91	73.6	161	18.7	33.0	3.31		27	1.31	1709		15.4	14.0	0.484
Orange River (iron)^1^	56	5.13	84.4	154	20.8	43.4	8.40		<200	0.63	10		0.128	4.7	1.072
Oroville	10	5.29	93.6	127	20.6	40.7	13.6		<200	0.39	<20		0.055	3.6	1.622
Owens Valley^1^	30	5.32	88.5	139	21.5	45.9	10.8		<150	0.46	<20		0.141	6.7	1.297
Picacho	147	4.92	71.9	184	18.3	32.9	3.38		<100	1.47	2250		21.1	17.0	0.456
Plymouth	68	5.24	83.6	154	21.9	42.4	7.56		<200	0.59	35		0.550	7.5	1.042
*Point of Rocks (iron)*	*100*	*5.08*	*82.8*	*166*	*20.7*	*41.2*	*5.76*	*6.1*	*<150*	*0.73*	*18*	*0.34*	*0.565*	*8.4*	*0.810*
Pontes e Lacerda^‡^	99	4.99	80.4	179	20.2	<50	5.28	8.8	<100	1.04	432	3.4	4.79	12.0	0.730
Poscente^1^	12	5.65	99.8	139	17.4	29.0	22.2	<1.2	136	0.18	<30	<0.15	0.022	1.5	2.308
Pozo Almonte^1^	32	5.11	86.9	148	22.1	43.8	8.15		79	0.59	41		0.238	5.1	1.058
Providence	42	5.18	83.6	161	20.6	41.5	6.49	5.0	<150	0.63	29		0.369	7.4	0.903
Quartz Mountain	181	5.01	77.5	161	19.7	36.0	4.99	11.6	<110	1.05	454		5.08	12.8	0.708
Quinn Canyon	21	5.21	86.2	162	20.7	41.5	7.36		<100	0.69	34		0.600	7.3	1.044
Rancho de la Pila (1882)	105	5.07	81.2	174	20.6	42.1	5.39		<120	0.78	50		0.778	8.9	0.794
Rancho Gomelia	13	5.65	97.4	141	17.3	28.7	21.5	<1.1	150	0.16	<40	<0.17	0.018	2.2	2.326
Rateldraai^1^	157	4.88	74.5	176	18.5	32.5	3.19		<200	1.26	2960		18.2	16.4	0.477
Red River^‡^	91	4.96	75.5	174	19.5	38.5	4.09	11.5	<150	1.11	453	3.5	5.10	13.8	0.614
Red Rock	30	5.04	78.1	161	20.5	41.8	6.70		67	0.90	179		2.30	11.3	0.816
Roebourne	92	5.08	81.3	166	20.9	42.4	5.56		<100	0.79	45		0.851	9.1	0.760
Roper River	22	5.62	98.3	149	18.6	33.9	18.5		<100	0.22	<15		0.020	3.6	2.049
Roundup^1^	35	5.11	84.9	168	21.3	42.2	6.85		50	0.75	70		0.871	8.6	0.892
Rowton	85	5.05	77.3	172	21.2	38.1	5.17	9.1	<150	1.00	262	2.1	2.89	12.1	0.703
Ruff's Mountain	28	5.18	85.8	121	21.2	46.9	9.44	4.5	<150	0.66	<50		0.517	7.1	1.083
Russel Gulch	277	4.92	75.3	166	19.2	35.6	3.76	11.2	<150	1.16	741	8.9	8.05	14.4	0.552
Sacramento Mountains	59	5.04	78.2	158	19.2	36.6	4.75		<100	1.11	648		7.09	12.4	0.675
Saint‐Aubin^‡^	14	5.63	103.3	130	17.1	<60	25.0	1.3	<200	0.20	<15		0.020	1.9	2.594
Samelia	105	5.02	79.2	175	20.5	38.3	5.20	10.2	<150	0.96	249	3.2	3.04	11.8	0.675
Sam's Valley	72	5.44	95.3	157	18.7	35.1	17.3		<250	0.31	<40		0.014	2.8	1.955
San Angelo	137	4.94	75.1	176	19.1	37.6	3.44	13.4	<300	1.50	869	11.0	9.34	13.4	0.521
Sanclerlandia	<100	4.90	74.4	173	19.0	36.4	3.73		35	1.19	667		7.04		0.574
Sanderson	38	5.62	94.4	137	18.2	35.9	16.2		<150	0.21	<30		0.025	3.2	1.850
Sandtown	21	5.16	82.3	161	20.4	41.4	6.42		<100	0.88	125		1.80	11.0	0.895
Santa Apolonia^1^	181	4.97	79.8	170	18.9	<91	3.78	12.0	<120	1.26	881	8.6	8.44	14.7	0.555
Savannah	25	5.10	79.3	157	21.0	44.0	6.09		<100	0.72	59		0.887	7.8	0.806
Schwetz	124	4.92	75.3	179	18.3	33.5	3.35	14.8	<150	1.24	1433	21.8	13.9	14.7	0.515
Seneca Falls	29	5.24	85.3	143	20.9	42.8	10.1		<120	0.50	20		0.324	4.3	1.190
Shandu [Hebei (iron)]	<40	4.95	76.3	157	19.6	36.9	4.13		48	0.97	592		6.39		0.602
Shişr 043^‡^	150	4.97	79.6	164	19.5	<80	4.31	10.4	<150	1.20	496	4.3	5.40	13.6	0.598
Sierra Sandon^1^	39	5.21	84.8	144	20.9	43.8	9.81		<130	0.87	<40		0.335	5.5	1.168
Slaghek's Iron^1^	14	5.16	86.6	144	21.8	51.0	8.50		90	0.73	35		0.448	8.9	0.997
Smith's Mountain	12	5.67	98.6	138	17.2	30.4	21.9		<160	0.22	<30		0.023	2.2	2.328
Spearman	21	5.23	86.7	117	20.3	46.0	8.87	5.3	<150	0.65	46	0.16	0.701	7.4	1.117
Ssyromolotovo	121	5.07	77.7	163	20.4	40.9	4.51		<200	1.12	338		4.20	13.6	0.619
Sterlitamak^1^	102	4.93	78.8	180	19.1	39.9	3.54		39	1.20	1120		9.77	14.5	0.563
Susuman	82	5.12	77.8	171	20.2	41.0	5.03		<200	0.95	174		2.33	11.2	0.687
Sychevka^1^	17	5.14	88.4	146	22.2		8.96			0.66	<40		0.413	5.5	1.082
Tagounite^1^	74	4.97	80.1	177	20.7	41.7	4.37		34	1.22	421		5.28	12.2	0.623
Tamarugal^1^	34	5.12	83.4	164	21.3	43.7	7.78		104	0.80	<60		0.587	6.6	1.056
Tambo Quemado	18	5.55	98.3	145	17.6	<50	20.1	1.2	103	0.16	<200		0.015	2.3	2.180
Tamentit^1^	31	5.18	85.2	144	20.8	42.7	8.29		<120	0.75	203		2.50	8.0	1.039
Tartak^‡^	111	5.00	73.8	176	19.2	<60	4.34	9.8	<150	1.02	380	2.7	4.16	13.1	0.612
Teplá	13	5.44	95.5	159	21.2	39.9	15.2		120	0.29	<30		0.016		1.762
Thunda	127	5.12	79.5	163	19.7	38.9	5.81	10.4	<150	0.87	191		2.78	11.2	0.811
Thurlow	11	5.67	101.1	126	16.2	27.3	22.6		<150	0.17	<30		0.019	2.2	2.374
Tieraco Creek	13	5.66	103.0	127	15.9	28.0	24.4		185	0.20	<50		0.045	5.2	2.565
Tonganoxie	75	4.94	77.5	165	19.6	38.5	4.64		<180	1.06	390		4.20	11.1	0.635
Toubil River	127	5.06	76.2	172	19.6	38.1	4.13		<130	1.09	474		5.39	12.5	0.582
Toubil River [Abakan]	84	5.02	74.9	164	19.6	42.3	4.28	9.9	<150	1.08	477	4.1	5.28	12.7	0.623
Trenton [location unknown]^1^	19	5.07	84.8	183	20.5	44.5	5.89		<300	0.93	190		2.42	10.1	0.822
Trenton [far from FeS]^‡^ ^,^ ^1^	71	5.07	80.5	155	20.4	<50	5.89	7.5	<150	0.94	201	1.9	2.50	9.6	0.815
Trenton [near FeS]^1^	32	5.24	84.5	149	21.0	<50	6.19		<100	0.84	192		2.26	8.8	0.844
Turtle River	80	5.27	89.8	135	21.0	41.4	10.6		<150	0.41	<30		0.071	4.8	1.274
Uruachic^1^	28	5.06	83.3	153	19.2	38.3	5.51		67	0.92	290		3.25	10.6	0.858
Uwharrie	95	4.96	77.5	160	20.1	38.9	4.68		<100	1.14	371		4.20	13.3	0.623
Veliko‐Nikolaevsky Priisk	13	5.21	86.5	125	21.0	47.3	8.73		<150	0.66	45		0.641	8.1	1.078
Verissimo	134	4.89	73.1	154	17.9	34.9	3.01		27	1.28	1906		15.2	18.0	0.487
Verkhne Udinsk	84	5.04	78.1	164	20.0	39.8	4.48		<200	1.09	332		3.93	12.6	0.631
Verkhnyi Saltov^‡^	45	5.00	79.3	144	20.1	<60	5.00	7.9	<110	1.13	264		3.20	12.2	0.691
View Hill	12	5.31	91.2	128	21.3	42.7	13.5		<200	0.44	30		0.318	5.0	1.568
Villa Regina^‡^	139	5.01	79.3	164	19.3	<70	4.30	10.1	<150	1.06	331		4.32	13.9	0.598
Wabar	71	4.98	74.6	168	19.2	38.4	3.82		<100	1.17	815		8.20	12.7	0.561
Wabar [Nejed]	100	4.98	74.3	165	19.2	37.9	3.96		<100	1.19	880		8.30	13.8	0.550
Waingaromia	11	5.28	92.3	147	21.1	41.6	10.8		116	0.55	37		0.381	6.8	1.413
Wallareenya^‡^	130	5.03	79.2	175	20.3	<50	4.15		<200	1.07	196		2.75	12.7	0.619
Welland	20	5.18	86.7	140	21.4	46.7	9.11		<70	0.57	17		0.349	8.4	1.196
Whitecourt^‡^	140	4.94	74.8	168	18.9	<50	3.66	12.2	<150	1.16	980	9.6	9.90	14.2	0.521
Williston^1^	115	4.91	76.6	177	18.8	33.8	3.41		31	1.37	1300		11.8	11.9	0.506
Wimberley	14	5.27	92.2	129	20.5	41.3	12.3		155	0.44	<20		0.149	5.3	1.511
Wisconsin Range 91614^‡^	122	4.98	75.7	172	18.6	<50	3.71		<200	1.19	730		7.91	14.6	0.580
Wolf Creek	14	5.37	93.3	143	19.5	37.3	13.9	2.4	<150	0.33	<20		0.028	3.4	1.587
Wonyulgunna	16	5.25	91.2	149	19.6	39.6	11.0		<200	0.35	<20		0.022	3.1	1.441
Yamato 790724^1^	127	5.02	76.2	181	19.4	35.9	3.41		31	1.21	970		9.32		0.554
Yarri	20	5.02	78.8	138	18.9	38.5	5.05	9.6	<150	0.90	434	5.5	5.37	12.3	0.770
York (iron)^1^	92	4.92	78.5	174	19.2	38.3	4.20		<150	1.16	473		5.53	10.2	0.623
Youanmi	65	5.12	81.2	165	20.6	37.4	5.60		<100	0.87	272		3.20	9.8	0.803
Zacatecas (1969)	14	5.47	97.4	107	20.0	38.8	16.2		<200	0.31	<70		0.031	2.8	1.937
Zerhamra^1^	66	4.96	78.8	172	19.4	33.5	4.47		<270	1.22	774		8.86	13.4	0.622
IIIAB‐an															
Delegate^1^	23	5.52	92.6	109	20.4	41.7	16.7		<600	0.62	196		1.77	8.2	1.727
Ilinskaya Stanitza^1^	12	5.52	92.1	114	19.7	39.2	16.7		<160	0.49	25		0.342	3.9	1.704
Palmas de Monte Alto^1^	14	5.37	92.8	136	20.4	<50	15.7	4.4	130	0.47	52	0.51	0.683	5.5	1.675
Petropavlovsk^§^ ^,^ ^1^	100	5.15	81.6	168	21.5	48.6	8.21	7.9	76	0.56	<180		0.575	8.7	0.962
Puente del Zacate^1^	49	5.04	80.8	158	19.7	40.5	4.88		<200	0.87	90		1.19	9.5	0.752
Treysa^1^	25	5.41	94.2	103	20.6	43.1	16.3		54	0.79	82		1.14	9.5	1.690
Yarovoye^1^	12	5.38	98.3	182	20.0	<50	15.4	4.6	<150	0.70	63	0.58	0.912	6.2	1.736
Ungrouped but related to IIIAB															
Elephant Moraine 92029^1^	18	5.06	82.2	123	23.8	<50	10.1		<100	0.34	<20		0.087	4.4	1.176
Ungrouped^¶^															
Ilimaes (iron) [FMNH]^1^	17	5.40	102.3	170	21.1	43.5	22.6		340	0.23	<50		0.132	5.3	2.520
Uegit^1^	483	5.46	79.2	168	16.8	33.9	3.21	16.8	<150	0.81	603		8.34	16.8	0.543
IAB‐ungr^¶^															
Marshall County^1^	17	5.94	78.6	113	21.1	44.4	15.2	12.6	<150	1.80	184	1.1	2.92	15.4	1.569

FMNH = Field Museum of Natural History, Chicago.

Italics: heavily weathered irons, Ider, Lucky Hill and Point of Rocks (iron), are not used for modeling or plotted in the figures.

[ ]—square brackets provide additional information for some irons, such as source or sampling location in the iron or synonyms.

^1^ See Table S1 for additional details of these analyses.

^‡^ Irons have not previously been listed in any UCLA publication.

^¶^ IIIAB irons in the Meteoritical Bulletin Database (MBDB) reclassified as other classes in this study.

^§^ Irons of other classes reclassified as IIIAB or IIIAB‐an in this study.

* Ge concentrations of Northwest Africa 4708 and Grein 005 are solely from INAA and may have large errors.

^2^ See Table S4 for individual Cape York analyses.

Over the course of the last 50 years, the UCLA team has analyzed up to 15 elements (Cr, Co, Ni, Cu, Ga, Ge, As, Ru, Sb, W, Re, Os, Ir, Pt, and Au, also Fe as the internal standard) in metals by instrumental neutron activation analysis (INAA). The analytical methods have been detailed in the literature (Wasson and Huber 2006; Wasson et al. 2007; Wasson and Choe 2009; Wasson 2011, 2016). The standards used were Filomena [North Chile] (IIAB), Coahuila (IIAB), and NBS809B (NBS steel). Some of the Ge and Sb data were determined by radiochemical neutron activation analysis (RNAA), and the upper limits for these elements come from INAA. The relative 95% confidence limits on the data in Table 1 are ≥10% for Cr; 1.5–3% for Co, Ga, and Au; 4–6% for Ni, As, Ir, and (RNAA) Ge (Wasson and Choe 2009; Wasson 2016). The confidence limits are 7–10% for concentrations higher than the following values (µg/g): (INAA) Ge, 150; Ru, 5; Sb, 0.20; W, 0.3; Re, 0.10; Os, 1.0; and Pt, 2 (Wasson 2011, 2016). The mean values of most specimens were calculated from multiple duplicates.

When calculating the mean values, analyses after 1986 (which had higher precision and accuracy) were given 1.5−2 times the weight of those before 1986. Since the NAA technique at UCLA has evolved over many years, John T. Wasson and his colleagues would frequently rerun previously analyzed irons along with also analyzing newly available iron meteorites. Of the meteorites listed in Table 1, data from UCLA analyses are reported for 42 samples for the first time. Analytical results from many of the irons listed in Table 1 have been previously published since 1986, but the compositional results were slightly revised after further efforts of John Wasson; Table 1 reflects these revised values, and Table S1 in supporting information provides additional details of the original publication of these irons, as well as some of John Wasson’s original notes on the pairings and compositional anomalies of some of the listed meteorites. Table S2 in supporting information details the reasoning for seven IIIAB irons that have been previously analyzed by the UCLA team not being listed in Table 1. The remaining currently identified IIIAB irons in the Meteoritical Bulletin Database (74 in total at the time of this study) have not been analyzed at UCLA.

Additionally, P data for IIIAB irons from Doan and Goldstein (1969), Moore et al. (1969), Lewis and Moore (1971), and Buchwald (1975) were used to include P in the modeling efforts; these previously reported P values are tabulated in Table S3 in supporting information. Cape York data are from Esbensen et al. (1982), Esbensen and Buchwald (1982), Buchwald (1987), and Table S4 in supporting information. Another set of irons considered in our modeling is the Treysa quintet, whose compositions are from Wasson (2016). Wasson (2016) suggested that the Treysa quintet formed from the same metallic core material as the IIIAB irons, and hence, we examine that formation hypothesis in our modeling in the next section.

## Modeling the IIIAB Group

The revised trapped melt model described in The Revised Trapped Melt Model section was applied to model the elemental trends in the IIIAB iron meteorite group. Partition coefficient parameterizations used for the modeling are given in Chabot et al. (2017). The next section describes the modeling results by dividing these elements into three main categories: strictly siderophile elements, schreibersite‐forming elements, and chalcophile elements. It is important to consider these groups of elements separately, as there are reasons why the elemental trends measured in IIIAB irons may not be fully reproduced for elements that partition strongly into schreibersite or troilite. These reasons are discussed in more detail in the sections below.

### Siderophile Elements: Co, Ga, Ge, As, Ru, Sb, W, Re, Os, Ir, Pt, Au

As shown in Fig. 2, the initial S content of the liquid metal has a large effect on the resulting crystallization trends for certain elements, and hence, this can be used to constrain the initial S content of the system. We iteratively explored different initial bulk compositions to provide the best fits to the IIIAB trends for the siderophile elements of Co, Ga, Ge, As, Ru, Sb, W, Re, Os, Ir, Pt, and Au. These 12 elements were selected to constrain the best fit of the model because these siderophile elements do not partition into phases such as schreibersite or troilite, and hence, their concentrations in the metal portion of IIIAB irons is expected to have been largely set by fractional crystallization and melt trapping. Figure 3 (Part 1), 3 (Part 2) shows the best fit achieved as constrained by these 12 elements, which uses an initial S content of 9 wt% and assumes that the lowest As IIIAB irons represent the first solids that crystallized from the fully molten core. The model in Fig. 3 (Part 1), 3 (Part 2) also includes 0.3 wt% P, which had a very minor influence on the elemental partitioning behavior in comparison to S, and the choice of that initial P content is discussed in Schreibersite‐Forming Elements: P and Ni section.

**Fig. 3 (Part 1):**
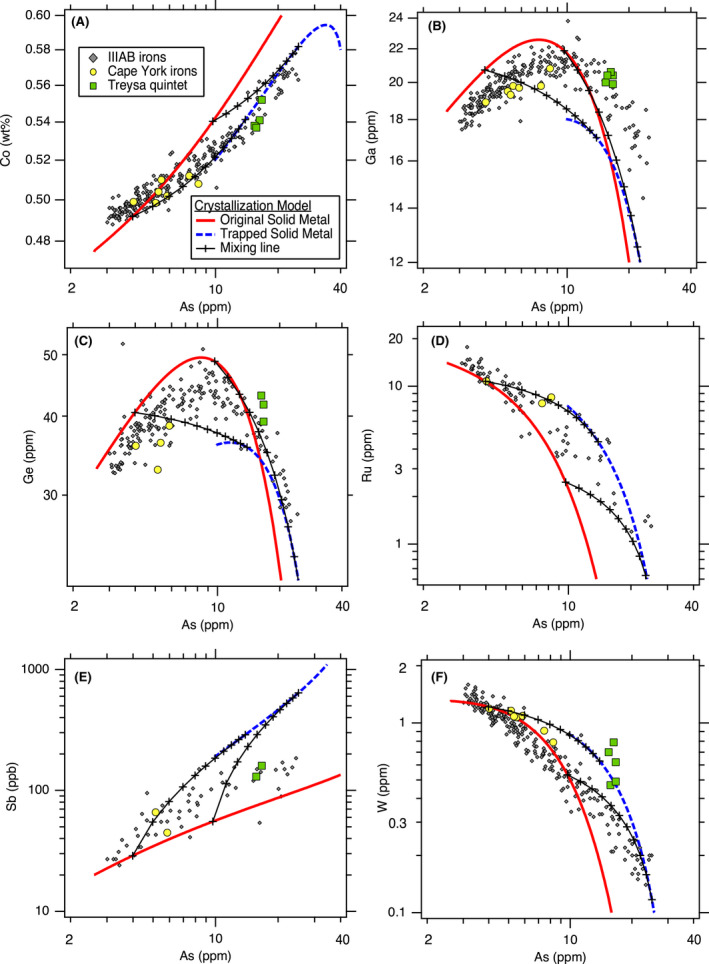
Revised trapped melt model applied to siderophile elements in the IIIAB group: (A) Co, (B) Ga, (C), Ge, (D) Ru, (E) Sb, (F) W versus As. See Fig. 3 (Part 2) for additional siderophile elements. The crystallization model results shown are for the preferred, best‐fit IIIAB model with an initial composition of 9 wt% S and 0.3 wt% P. The two mixing lines are shown at 28% and 56% crystallization.

**Fig. 3 (Part 2):**
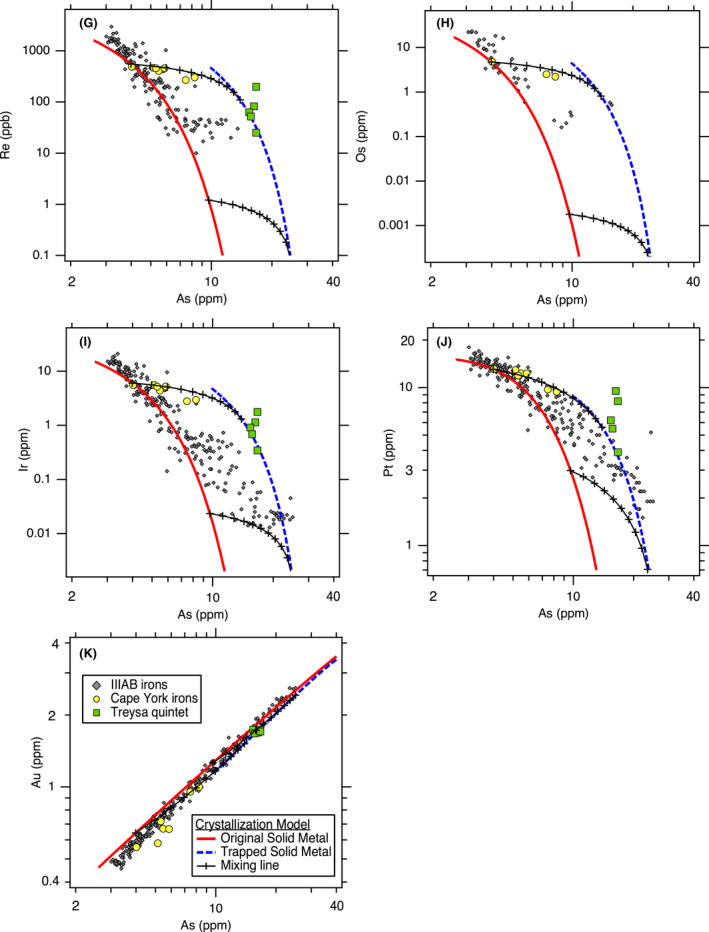
Revised trapped melt model applied to siderophile elements in the IIIAB group: (G) Re, (H) Os, (I) Ir, (J) Pt, and (K) Au versus As. See Fig. 3 (Part 1) for additional siderophile elements. The crystallization model results shown are for the preferred, best‐fit IIIAB model with an initial composition of 9 wt% S and 0.3 wt% P. The two mixing lines are shown at 28% and 56% crystallization.

The original trapped melt model of Wasson (1999) examined the IIIAB Ir trend versus both As and Au, with similar results for the choice of either As or Au. In the Wasson (1999) results, the compositions of the IIIAB irons are located between the model curves of the solid metal and liquid metal that formed during fractional crystallization, leading to the interpretation that IIIAB irons formed by fractional crystallization with various amounts of trapped melt by individual specimen to explain the scatter in the IIIAB data. On Fig. 3I, we show the Ir versus As results for this revised trapped melt model as applied to the IIIAB irons, and the plot is very similar in appearance to the plots in Wasson (1999). In Fig. 3I, the IIIAB iron data largely fall between the solid metal curve and the curve from solid metal that forms as a result of trapped melt. However, while Fig. 3I strongly resembles the figures in Wasson (1999), there are two very important differences: (1) Fig. 3I uses partitioning behavior for Ir that is consistent with the experimental data, unlike that used by Wasson (1999) and shown on Fig. 1. (2) The curve shown in Fig. 3I is not the liquid metal curve, which is what Wasson (1999) used, but rather is the solid metal that forms from the trapped liquid, as described in Equations ((6), (7), (8)). With these mathematical adjustments in this revised trapped melt model, the trapped melt concept of Wasson (1999) is still able to explain the IIIAB Ir versus As trend in Fig. 3I in a manner similar to that originally envisioned.

Overall, the results in Fig. 3 (Part 1), 3 (Part 2) show that the revised trapped melt model is able to fit the general IIIAB trends for the 12 elements examined and also provide an explanation for the scatter in the IIIAB data as due to different amounts of trapped melt. This is an important accomplishment because no previous IIIAB trapped melt modeling efforts have simultaneously modeled 12 elements with one consistent model. In the original trapped melt model of Wasson (1999), and the follow‐on study of Wasson and Richardson (2001), only Ir, As, and Au were modeled. Wasson and Choi (2003) also included Ge and Ga in their IIIAB modeling efforts, in addition to Ir, As, and Au. Wasson (2016) modeled Ir, W, and Au for the IIIAB group. Thus, previous trapped melt models had been applied to six elements total in the IIIAB group but with inconsistent partitioning behaviors utilized from study to study, as illustrated in Fig. 1 for *D*(Ir).

The success of this revised trapped melt model in fitting 12 elements simultaneously in one consistent model, as shown in Fig. 3 (Part 1), 3 (Part 2), provides strong support to the conceptual idea first presented by Wasson (1999) that trapped melt played an important role during the crystallization of IIIAB irons. The revised trapped melt model presented here differs in the mathematical implementation from the model of Wasson (1999), by accounting for the formation of troilite, which is not included in the elemental analyses of the meteorites plotted in Fig. 3 (Part 1), 3 (Part 2). However, conceptually, the trapped melt model of Wasson (1999) is unchanged, where melt is envisioned to be mechanically trapped during crystallization, followed by diffusional leveling of compositional gradients as the melt solidifies. As suggested by Wasson (1999), the lack of compositional gradients in most IIIAB irons, even those that plot as having substantial amounts of trapped melt, implies that melt must have been trapped on a small scale, with roughly meter‐scale or less distances.

Looking at the details in Fig. 3 (Part 1), 3 (Part 2), one can see the fits are imperfect. Looking only at Ir versus As, one might conclude that a slightly higher initial S content of 10 wt% does a better job at fitting the trend. In contrast, looking only at Ge or Ga versus As, one might conclude that a slightly lower initial S content of 8 wt% S is actually preferred. Figures S1–S3 in supporting information provide the model results using initial S contents of 8, 9, and 10 wt% S, respectively, so that the subtle differences in the model as a function of S content can be compared. Figure 1 shows that although the experimental data for *D*(Ir) as a function of liquid metal S content exhibit a clear trend, there is scatter among the experimental data, such that one could envision a slightly different *D*(Ir) fit that was still consistent with the experimental results. A slightly different parameterized dependency of *D*(Ir) on the liquid metal S content, or on how the effects of S and P should be combined (Chabot et al. 2017), will result in slight differences in the model curves shown on Fig. 3 (Part 1), 3 (Part 2) and in Figs. S1–S3. Thus, minor discrepancies in Fig. 3 (Part 1), 3 (Part 2) should not be interpreted as a failure of the concept of the model but rather as a limitation of the precision with which we know the partitioning behavior of each element as a function of the evolving liquid metal composition and variations between these natural samples due to sample size or other processes. Overall, the fact that 12 elements are fit successfully for the IIIAB irons with a single model that utilizes experimentally determined partition coefficients and that indicates a consistent initial IIIAB S content for all the elements modeled provides strong credibility and confidence in the revised trapped melt model approach.

Two examples of mixing lines between the solid metal formed originally from fractional crystallization and the solid metal formed by the solidification of trapped melt are shown on Fig. 3 (Part 1), 3 (Part 2) The mixing line shown at 56% crystallization is roughly consistent with the extent of the IIIAB trends. The percent crystallization refers to the percent of the total core by mass that has solidified; the model begins with a 100% liquid core at 0% crystallization. The 56% crystallization is based on the assumption that the lowest As IIIAB irons represent the first solids to form from a fully molten core. For the IIIAB group, which has hundreds of members that define the IIIAB trends, this assumption of the initial crystallization products of the core being sampled by the IIIAB irons seems reasonable in comparison to an assumption that the initial solids formed in the IIIAB core are missing from our meteorite collections. However, it is important to note that from a modeling perspective alone, these two scenarios cannot be distinguished, as the amount of crystallization and the S content of the liquid are correlated.

The limited extent of the IIIAB trends for Re and Os shown in Fig. 3 (Part 2) are consistent with irons that would be expected to have low Re and Os values having concentrations for these elements that are below detection limits. The IIIAB irons with the lowest Ir values in Table 1 lack corresponding measurements for Re and Os. Thus, the Re and Os IIIAB trends are an incomplete sampling of the extent of core crystallization. A crystallization of 56% means that as the temperature decreases and fractional crystallization of the core proceeds, 56% of the core is solid metal at this point. For a starting 100% liquid composition of 9 wt% S, the liquid metal composition at the point of 56% crystallization has roughly 20 wt% S. The eutectic in the Fe‐FeS system is at ~31 wt% S, and the presence of Ni shifts the formation of troilite to slightly lower S contents but only to ~29 wt% S at the Ni content of IIIAB irons (Raghavan 2004).

One explanation of the IIIAB trend only extending to 56% crystallization is that fractional crystallization may have proceeded to higher S contents and is just not sampled by the IIIAB irons we have as meteorites. However, the fact that there are over 300 meteorites classified as IIIAB irons is a challenge for this explanation that implies the IIIAB trend is incompletely sampled. Alternatively, the fact that the IIIAB irons do not extend further along the fractional crystallization solid metal trends in Fig. 3 (Part 1), 3 (Part 2) may indicate that fractional crystallization ceased before the cotectic composition in the Fe‐Ni‐S was reached. Figure 3 (Part 1), 3 (Part 2) shows that the compositions of many IIIAB irons are best explained by containing a substantial amount of solid metal that formed by solidification of trapped melt. A possible scenario is that when 56% of the core had solidified, the 44% of the core that remained as liquid metal was actually in trapped melt pockets that had been trapped throughout the crystallization sequence. These trapped melt pockets eventually solidified, resulting in the solid metal formed from trapped melt curves shown on Fig. 3 (Part 1), 3 (Part 2). Thus, while the fractional crystallization trend only extends to 56% crystallization, the other 44% of the IIIAB core may not be missing from our collections entirely but rather may be represented by the IIIAB irons that show a large trapped melt component.

However, even in this scenario, there is still a sizable amount of S‐rich material that is largely missing from our meteorite collections. An initial 9 wt% S liquid composition will produce roughly 29% troilite and 71% Fe‐Ni metal by mass, and IIIAB irons do not contain troilite at these high levels. In this scenario, an additional explanation is still needed to explain the missing troilite, such as being weaker and hence more easily destroyed and underrepresented in our meteorite collections (Kracher and Wasson 1982). An alternative hypothesis is the process of “ferrovolcanism,” where S‐rich melts from the core are buoyantly propagated via dykes into the mantle, with the possibility of erupting on the surface of the asteroid (Johnson et al. 2020). Johnson et al. (2020) describe the process of ferrovolcanism as being well suited to asteroid cores that crystallize from the core–mantle boundary inward with a relatively thin mantle. The range of cooling rates of IIIAB irons support such inward crystallization of a parent body core that lacked a fully insulating silicate layer (Yang and Goldstein 2006; Goldstein et al. 2009), so the option of ferrovolcanism being responsible for the removal of S‐rich melt from the crystallizing IIIAB core is consistent with the conditions presented for this scenario.

The second mixing line shown on Fig. 3 (Part 1), 3 (Part 2) is at roughly 28% crystallization, at which time the liquid metal has a composition of about 12.5 wt% S. This mixing line represents one example to explain the compositions of the suite of Cape York irons (Esbensen et al. 1982), which also are plotted in Fig. 3 (Part 1), 3 (Part 2). The Cape York irons offer unique insight into the crystallization of the IIIAB parent body, given they represent 57 tons of recovered mass that display compositional variations across the samples and large troilite nodules interpreted to be formed by trapped melt during core solidification (Esbensen and Buchwald 1982; Esbensen et al. 1982). The modeling results in Fig. 3 (Part 1), 3 (Part 2) show that the Cape York iron siderophile element compositions in the Fe‐Ni metallic phase are consistent with those predicted by the revised trapped melt model for containing different contributions of a trapped melt component. As discussed before, there are slight discrepancies between different elements, consistent with the limitations of understanding the partitioning behavior of each element as a function of the evolving metallic liquid composition and the variability between natural samples. In particular, picking a mixing line at a lower percent of crystallization can produce a better fit to the Cape York Ga compositions in Fig. 3B but would provide a poorer fit to the Ir compositions in Fig. 3I. Overall, the revised trapped melt model results in Fig. 3 (Part 1), 3 (Part 2) support the conclusion that the siderophile element variations between the Cape York specimen are due to different amounts of a trapped melt component contributing to the different specimens.

Another group of irons plotted on Fig. 3 (Part 1), 3 (Part 2) are the Treysa quintet. Wasson (2016) suggested that the composition of these IIIAB irons were formed by mixtures of solid metal that formed after 3% crystallization and trapped melt from much later during fractional crystallization. This conclusion was supported by modeling the trends of the Treysa quintet for Ir and W versus Au. Looking at Fig. 3 (Part 1), 3 (Part 2), one can see that such an explanation could also be consistent with the revised trapped melt model results for these three elements. However, when additional elements are considered, in particular Ga and Ge, it is clear that this hypothesis is not able to explain these compositions. The Treysa irons have Ga and Ge contents that are higher than IIIAB irons with the lowest As, which are the first solids to form by the crystallization sequence. Solid metal that forms from a late‐stage liquid has very low Ge and Ga contents. Thus, there is no way that mixing between the earliest stage solid metal and the later stage trapped melt could produce the high Ga and Ge contents present in the Treysa quintet irons. Wasson (2016) did not evaluate how his proposed model would explain the high Ga and Ge contents of these irons, but when these elements are considered, the proposed explanation that Treysa irons are mixtures of early solids and late trapped melt does not seem viable. Similar to the Terysa quintet, main group pallasites similarly plot near the IIIAB irons on many siderophile element trends but have higher Ga and Ge contents than either early or late formed IIIAB irons (Wasson and Choi 2003). Wasson and Choi (2003) also concluded that the Ga and Ge contents of main group pallasites could not be formed by mixing early and late materials from the IIIAB core and considered alternative hypotheses related to formation in a body more enriched in Ga and Ge than the IIIAB core or compositional effects resulting from an impact mixing formation event. Whether main‐group pallasites are from the same parent body as IIIAB irons continues to be debated, given their cooling rates do not seem consistent with formation in the same asteroid as IIIAB irons (Yang et al. 2010) but their oxygen (Clayton 2003) and sulfur isotopes (Dottin et al. 2018) match those of IIIAB irons, along with the similarities in the metallic compositions. Further work that uses the revised trapped melt model to examine potential implications for the formation of main group and other pallasites is worthwhile.

The Treysa irons are still very interesting to consider, given their unusual high As and high Ir, Pt, and W compositions. As shown in Fig. 3 (Part 1), 3 (Part 2), the revised trapped melt model suggests that these irons are formed dominantly by solid metal that formed from trapped melt and not directly by fractional crystallization. The Ga and Ge contents are still too high for this explanation using the 9 wt% S model shown in Figs. 3B and 3C, but the behaviors of these elements are sensitive functions of the S content of the metallic liquid. In fact, examining the alternate fits produced with an initial starting composition of 8 wt% S and shown in Fig. S1, the Treysa irons fall closer to the curve expected for solid formed from a trapped melt for both of these elements. If the Treysa irons formed from a large region of trapped melt, it is possible that the variations among those irons might be due to the method by which that pool of trapped melt solidified, which could be worth more detailed consideration by a future study.

There are a few final observations worth making about Fig. 3 (Part 1), 3 (Part 2). First, Sb has the potential to be a highly diagnostic element to indicate formation from a trapped liquid. Experimental studies have shown that in the Fe‐Ni‐S system, Sb displays siderophile rather than chalcophile behavior and that *D*(Sb) remains <1 over the full range of S contents up to the Fe‐S eutectic composition (Chabot et al. 2017). Thus, any liquid trapped during crystallization will have a higher Sb content than the corresponding solid metal, and assuming that no Sb partitions into the troilite that forms, the solid metal that forms from the trapped liquid will be even further enriched in Sb. Figure 3E shows the consequence of this unique partitioning behavior, where Sb values are modeled to be an order of magnitude higher for solids that formed from a trapped melt versus those that formed directly from fractional crystallization. Measurements of Sb are more limited in iron meteorites than measurements for many other trace elements, but this result provides strong motivation for future studies to include Sb measurements whenever possible when analyzing iron meteorites. Additionally, the parameterization of *D*(Sb) in Chabot et al. (2017) has some of the largest errors of all the elements examined, and given the modeling results in this study, future efforts to improve the knowledge and parameterization of the partitioning behavior of Sb would be worthwhile.

Last, the fit to the IIIAB Co trend suggests that the Co trend is more nuanced than previous modeling efforts have appreciated. For example, the Co versus As trend (Fig. 3A) is largely linear, as is the Au versus As trend (Fig. 3K). However, the revised trapped melt model fits to these two linear trends are very different. For the Au versus As trend, the solid metal formed from fractional crystallization and the solid metal formed from trapped melt form the same line, as do mixing lines between the two consequently. In contrast, for Co, the solid metal formed from fractional crystallization and the solid metal formed from trapped melt trends do not overlap, and to match the Co trend of IIIAB irons, a trapped melt component is required. This is completely consistent with the conclusions drawn from other elements, such as Ir, that trapped melt was an important process during the crystallization of the IIIAB core, but it has not been appreciated previously that modeling Co in iron meteorites might also be diagnostic. However, as seen in Fig. 3A, the variation in Co over the entire range of IIIAB irons is minimal, which still limits its usefulness to distinguish models in comparison to an element like Ir that exhibits order of magnitude variations, but this new understanding of the IIIAB Co trend is still worth noting here.

### Schreibersite‐Forming Elements: P and Ni

With any model, it is important to understand its limitations. This revised trapped melt model does not include the formation of schreibersite. Schreibersite is known to occur in IIIAB irons (Buchwald 1975) by subsolidus exsolution of P from the solid metal (Doan and Goldstein 1969; Clarke and Goldstein 1978). Subsolidus growth of schreibersite influences the P and Ni contents of the solid metal from which it forms, as P and Ni are major components in the schreibersite. The subsolidus growth of schreibersite uses P and Ni from the solid metal to create the schreibersite, leaving the resulting solid metal depleted in P and Ni relative to the composition of the solid metal that crystallized from a liquid. Measurements of the solid metal of IIIAB irons thus reflect the solid metal composition after subsolidus schreibersite formation has occurred. In contrast, the revised trapped melt model tracks the composition of the solid metal as it solidified from a liquid, prior to subsolidus schreibersite formation. Consequently, the concentrations of any elements in the IIIAB irons that have been significantly affected by the formation of schreibersite, or any other subsolidus redistribution process, should not be expected to be fully fit by the revised trapped melt model. In particular, this includes the elements of P and Ni, as well as trace elements with a strong affinity for schreibersite, such as Mo (Chabot et al. 2020). If iron meteorite measurements were bulk composition measurements, with the present‐day Fe‐Ni metal and the schreibersite phases integrated into one compositional value, then the revised trapped melt model would be predicted to match this compositional value; in practice, such bulk measurements are rarely made and are limited by sampling bias, given the size of most meteorite samples and the coarse distribution of large schreibersite phases.

While fully appreciating this limitation, we do apply the revised trapped melt model here to the P and Ni IIIAB trends because (1) the P content of the metallic liquid influences the partitioning behavior and thus providing any constraint available on that initial IIIAB liquid metal P content is worthwhile and (2) the Ni trend has been investigated with previous crystallization models, making it important to discuss directly.

**Fig. 4:**
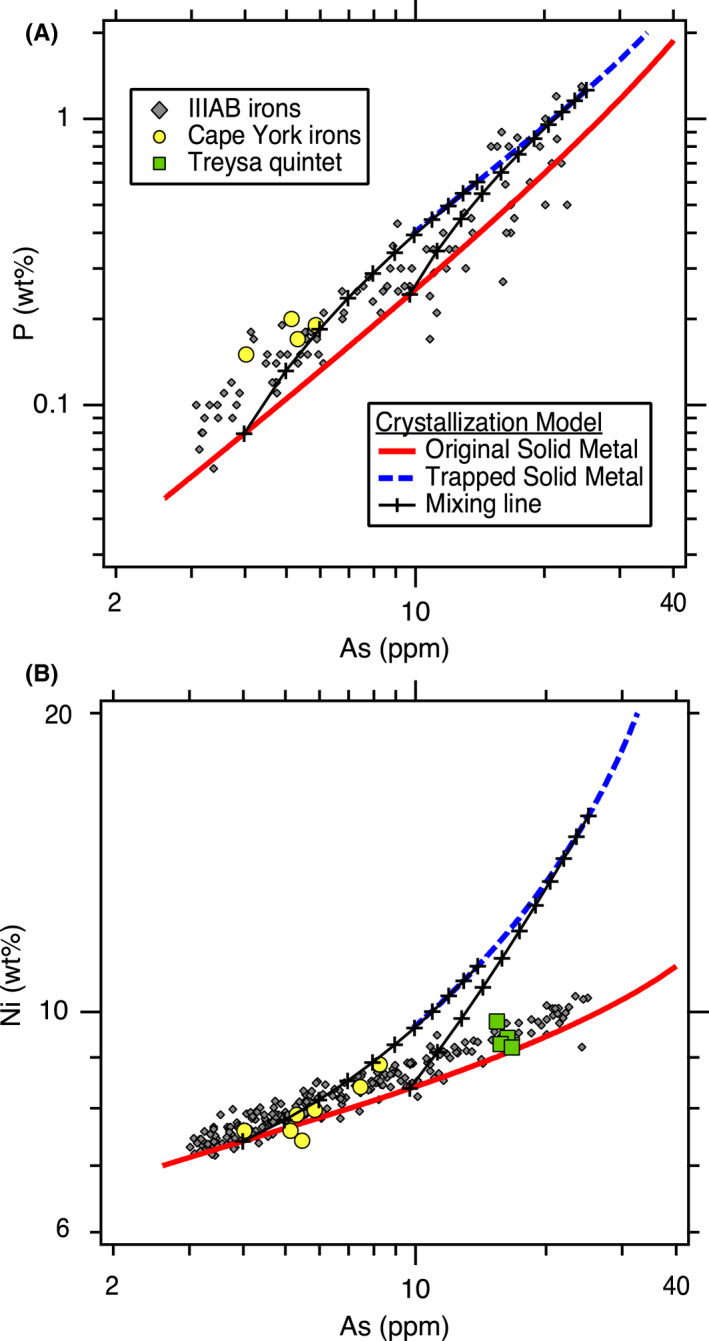
Revised trapped melt model applied to (A) P and (B) Ni in the IIIAB iron meteorite group. The concentrations of P and Ni in the metal of IIIAB irons can be affected by the formation of subsolidus schreibersite, and the revised trapped melt model does not include effects due to schreibersite formation or other subsolidus processes that occurred subsequent to solidification of the metal. The crystallization model results shown are for the preferred, best‐fit IIIAB model with an initial composition of 9 wt% S and 0.3 wt% P. The two mixing lines are shown at 28% and 56% crystallization.

Figure 4A plots IIIAB P data compiled from estimates and measurements by Doan and Goldstein (1969), Moore et al. (1969), Lewis and Moore (1971), and Buchwald (1975), which are tabulated in Table S3. As discussed in Scott (1972), some of these P estimates involve attempts to correct for and include the presence of macroscopic schreibersite, but other P values were obtained from samples that attempted to avoid large schreibersite nodules and to sample only the Fe‐Ni solid metal phase. The solubility of P in solid Fe‐Ni metal decreases as the solid metal cools, leading to the exsolution of P and the formation of schreibersite at subsolidus conditions (Raghavan 1988; Okamoto 1990). Thus, the P concentrations measured in the solid metal phase of IIIAB irons currently are expected to be lower than the P concentrations of the solid metal when they first solidified in the IIIAB parent body. The P values plotted in Fig. 4A are not necessarily the bulk P concentrations of these samples, as even those that account for the presence of large schreibersites are based on the assumption that the meteorite sample is representative of the bulk abundance of schreibersite. Applying the trapped melt model to the IIIAB P trend in Fig. 4A provides a lower limit to the initial P content of the metallic liquid, estimated to be 0.3 wt% P for the best‐fit model with 9 wt% S.

This initial P content is used, along with the *D*(P) parameterization of Chabot et al. (2017), to track the evolution of P in the metallic liquid throughout crystallization. The P content of the liquid metal also influences the partition coefficients of other trace elements, as captured by the parameterizations in Chabot et al. (2017) and the equations in The Revised Trapped Melt Model section. While the P content of the liquid does influence the partitioning behavior, for the IIIAB modeling, it is a minor effect in comparison to S. The initial S concentration is estimated to be substantially higher than that of P, 9 wt% S versus 0.3 wt% P; even if that P content is underestimated, it would not approach a value comparable to the S estimate. Additionally, S is insoluble in the solid metal that forms, resulting in the liquid metal quickly evolving to highly enriched S contents while P partitions partially into the solid metal that forms and does not become as highly enriched in the liquid metal as crystallization proceeds. Liquid immiscibility in the Fe‐Ni‐S‐P system can lead to two liquids, one S‐rich and one P‐rich (Ulff‐Møller 1998; Chabot and Drake 2000), but those effects are beyond those considered in this revised trapped melt model. Overall, applying the revised trapped melt model to the available IIIAB solid metal P data supports an initial P content of at least 0.3 wt% P, and this initial P content is included as a minor influence on the partitioning behaviors of the elements modeled in this study.

Nickel is a major component of meteoritic schreibersite, and additionally, as the temperature decreases, the Ni content of the schreibersite continues to increase through the process of solid‐state diffusion (Doan and Goldstein 1969; Clarke and Goldstein 1978). Thus, the measured Ni contents in the solid metal compositions of present‐day IIIAB irons have been affected by the formation of schreibersite, and any model that does not consider that schreibersite formation should not be expected to fully match these measured IIIAB solid metal Ni values. Nevertheless, the result of applying the revised trapped melt model to the IIIAB Ni trend is shown in Fig. 4B.

A striking feature of Fig. 4B is how little scatter in the IIIAB Ni values there is and how closely they follow the solid metal formed by the fractional crystallization curve in Fig. 4B. This is in notable contrast to many elements in Fig. 3 (Part 1), 3 (Part 2), such as Ir, which show substantial scatter between the solid metal formed by fractional crystallization and solid metal formed from trapped melt curves. The IIIAB meteorites plotted in Fig. 3 (Part 1), 3 (Part 2) and 4B are the same, from Table 1. Thus, if solid metal formed from trapped melt is an important component of IIIAB irons, that conclusion would be expected to be consistent for all elements, and the fact that the IIIAB Ni trend does not show evidence for substantial components that formed by trapped melt would be a concern. However, the revised trapped melt model does not consider the formation of schreibersite, or other subsolidus redistribution processes, which may have influenced the solid metal Ni concentrations of IIIAB irons subsequent to their solidification.

The fact that the IIIAB Ni trend does not show compositions with high trapped melt contributions may indicate that irons that formed from substantial amounts of trapped melt also subsequently formed more schreibersite. Trapped melt is enriched in P, which would form schreibersite as that trapped melt solidified, which would draw Ni out of the solid metal and into the schreibersite phase. This can cause a meaningful change in the Ni content of the metal, such as displayed in the IIG group relative to the IIAB group. There is compelling evidence that IIG irons are related to late‐stage IIAB irons, though their Ni contents differ by ~2 wt%, which is attributed to the P‐rich nature and prevalent schreibersites of IIG irons (Wasson and Choe 2009; Chabot et al. 2020). Thus, meteorites with a higher trapped melt content would be expected to be enriched in P and have higher amounts of schreibersite exsolution. Additionally, trapped melt may experience liquid immiscibility in the Fe‐Ni‐S‐P system as it solidifies (Ulff‐Møller 1998) which could influence the composition; however, the fact that this revised trapped melt model works well for many elements without including this effect suggests such a process may not have a large influence, but it is worthy of future study. That the Ni trend shows so little scatter, in contrast to many of the other elements examined in this study, may support that the subsolidus schreibersite formation process is contributing to the leveling of Ni values across the meteorite sample, and this topic is worthy of more detailed examination by future studies.

Overall, crystallization models that do not consider effects of schreibersite formation can be applied to the P and Ni trends in magmatic iron meteorites to provide some basic insight into the bulk initial compositions of these elements. However, fitting these elements should not be used to constrain or evaluate any models that do not also quantitatively consider the effects of schreibersite formation. Previous fractional crystallization models have modeled Ni trends in magmatic irons (e.g., Jones and Drake 1983; Haack and Scott 1993; Ulff‐Møller 1998; Chabot and Drake 1999), but it is important that future models also consider the effect of schreibersite on the measured solid metal Ni contents if such trends are to be fully explained.

### Chalcophile Elements: Cr and Cu

Another limitation of the revised trapped melt model as implemented in this study is for applications to chalcophile elements. The assumption made in Equation (8), that the element does not partitioning into troilite, is not necessarily valid for chalcophile elements. If an element does partition into troilite, then Equation (6) could be used in a manner that includes that partitioning behavior, but that is beyond the implementation in this first study. Thus, this revised trapped melt model should not be applied to chalcophile elements without considering their potential to partition into the troilite that forms from trapped melt.

**Fig. 5:**
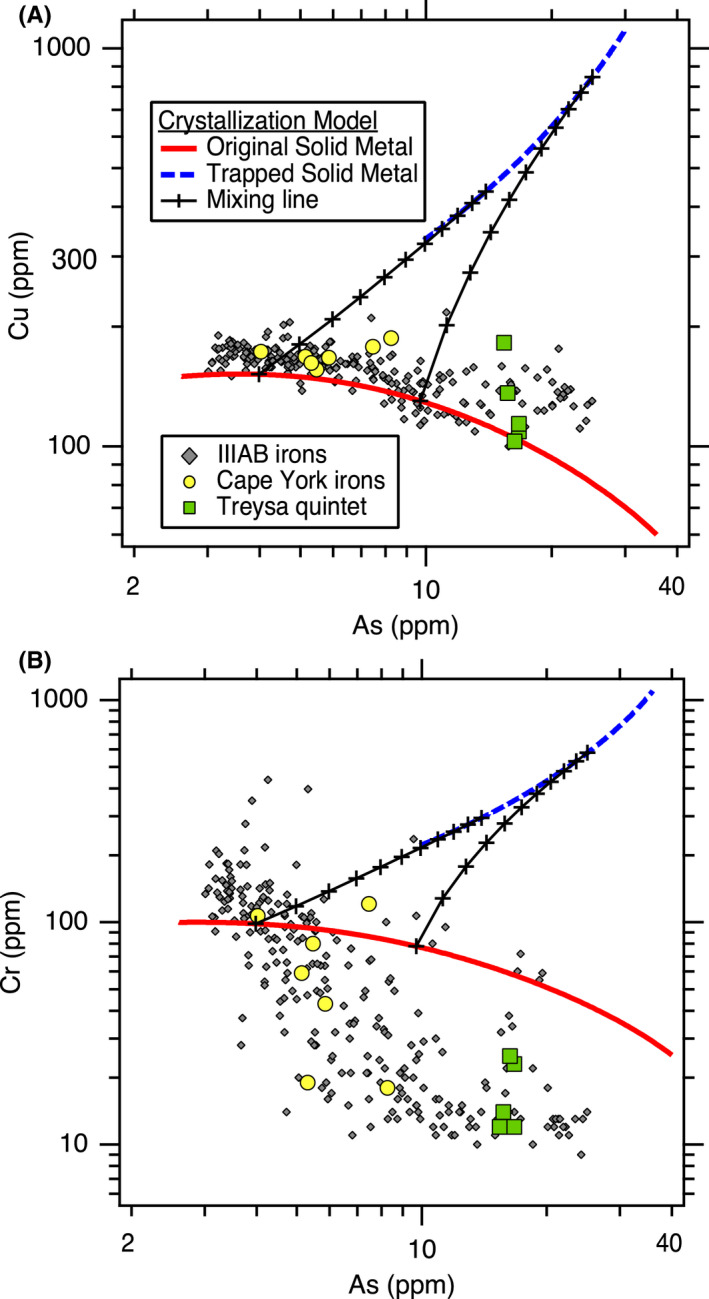
Revised trapped melt model applied to (A) Cu and (B) Cr in the IIIAB iron meteorite group. Both Cu and Cr are chalcophile and may partition into troilite, and that effect is not included in the revised trapped melt model shown here. The crystallization model results shown are for the preferred, best‐fit IIIAB model with an initial composition of 9 wt% S and 0.3 wt% P. The two mixing lines are shown at 28% and 56% crystallization.

Table 1 reports IIIAB data for Cu and Cr, and thus, it is worthwhile to apply the revised trapped melt model to these elements to see the outcome. Figure 5A plots the IIIAB Cu trend, and the revised trapped melt model does a good job of matching the general trend. However, like the Ni trend in Fig. 4B, the Cu trend shows little evidence for requiring substantial amounts of a trapped melt component, in contrast to elements such as Ir in Fig. 3I. This could be because a solid metal that formed from solidification of a trapped melt would also form more troilite, which could sequester Cu given its chalcophile nature. The revised trapped melt model appears adequate to help constrain the initial bulk Cu content of the IIIAB core but should not be expected to fully explain the IIIAB Cu trend without adjusting the model to also include the partitioning of Cu into troilite.

Figure 5B shows the revised trapped melt model applied to the IIIAB Cr trend, and the model fails to adequately reproduce the IIIAB solid metal measurements. The IIIAB Cr trend has considerable scatter and large variations of a factor of 10 among different IIIAB irons. Wasson (1999) suggested that the unusual IIIAB Cr trend could be due to chromite grains, both as a sampling artifact where such grains are avoided and because such grains would sequester Cr in a manner beyond that considered in the fractional crystallization models. Chabot et al. (2009) investigated the partitioning behavior of Cr and also concluded that the formation of chromite was a likely explanation for their inability to model the IIIAB Cr trend by fractional crystallization. Overall, the IIIAB Cr trend is fit so poorly in Fig. 5B, that it is not even adequate to provide a meaningful constraint on the initial bulk Cr content of the IIIAB core.

### Bulk IIIAB Core Composition

Table 2 summarizes the initial compositions used in the revised trapped melt model for our preferred fit with an initial concentration of 9 wt% S, as shown in Figs. 3 (Part 1), 3 (Part 2), 4, 5. The concentration of Fe is not modeled independently but calculated at each step in the model based on the composition of each phase totaling 100%. As discussed in the Siderophile Elements: Co, Ga, Ge, As, Ru, Sb, W, Re, Os, Ir, Pt, Au section, some elements such as Ir are fit slightly better with a higher S content of 10% (Fig. S3) while other elements such as Ga and Ge are fit slightly better with an initial S content of 8 wt% (Fig. S1). These discrepancies are attributed to the limitations of the precision with which we know the partitioning behavior of each element as a function of the evolving liquid metal composition. Producing model results for these three different S contents provides a mean to quantify the uncertainties in the initial IIAB core composition, as the S content has a larger influence on the partitioning behavior than the uncertainties in the best‐fit parameterizations (Chabot et al. 2017). Also, all three models make the assumption that the lowest As IIIAB irons represent the first solids formed by solidification of a fully molten IIIAB core. If the early portion of the IIIAB core is missing from our meteorite collections, then the initial compositions of the models would need to be adjusted, but there is no evidence to suggest we are missing early crystallized IIIAB irons. The results in Table 1 show that the three different models yield bulk compositions that are generally within 10% of each other, showing good general agreement in the overall bulk composition to this level of certainty.

**Table 2:** Modeled bulk composition of the IIIAB parent body metallic core.

Element	CI	Condensation *T* (K), Wood et al. (2019)	8 wt% S model	9 wt% S model	10 wt% S model
P (wt%)	0.097	1287	0.35	0.30	0.30
S (wt%)	5.35	672	8.0	9.0	10.0
Fe (wt%)	18.5	1138	83.9	83.0	81.9
Co (wt%)	0.051	1354	0.41	0.39	0.38
Ni (wt%)	1.08	1363	7.36	7.27	7.38
Cu (ppm)	131	1034	239	248	268
Ga (ppm)	9.71	1010	14.6	13.6	12.8
Ge (ppm)	32.6	830	29.3	27.1	25.6
As (ppm)	1.74	1235	8.4	7.4	7.9
Ru (ppm)	0.686	1533	5.8	5.7	5.5
Sb (ppb)	145	890	173	143	168
W (ppm)	0.096	1736	0.73	0.66	0.59
Re (ppb)	39.3	1736	379	358	351
Os (ppm)	0.493	1806	3.6	3.4	3.5
Ir (ppm)	0.469	1566	3.0	3.7	2.8
Pt (ppm)	0.947	1370	7.4	6.7	6.0
Au (ppm)	0.146	967	0.90	0.87	0.86

**Fig. 6:**
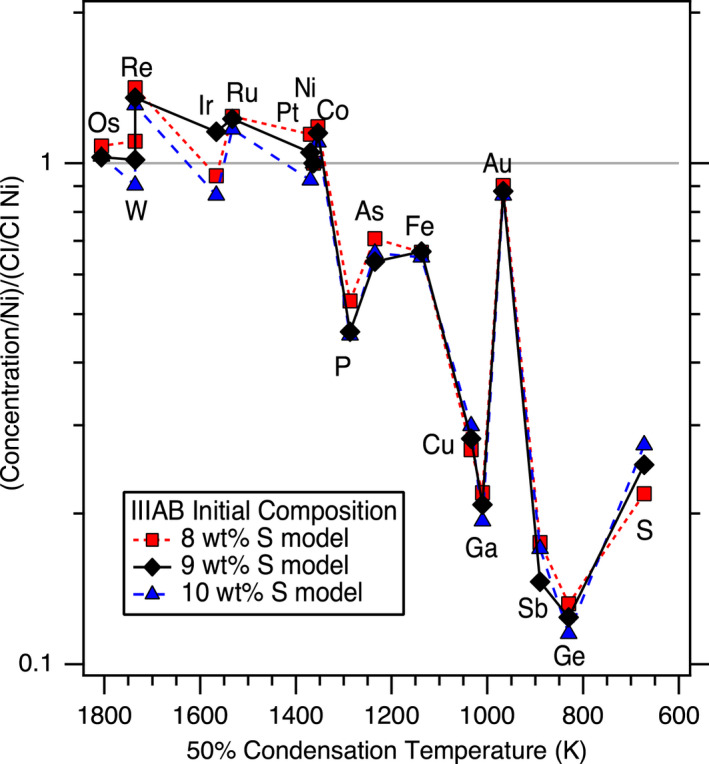
Bulk composition of the IIIAB core as determined from the revised trapped melt model. The model with an initial composition with 9 wt% S is the preferred best‐fit model, and the range of solutions shown from 8 to 10 wt% S illustrates the uncertainty associated with the best‐fit determination.

Figure 6 plots the bulk compositions from Table 2, normalized by Ni and CI chondrite compositions (Lodders 2010), as a function of their 50% condensation temperature reported in Wood et al. (2019). The siderophile elements in Fig. 6 with 50% condensation temperatures greater than ~1300 K have modeled bulk IIIAB core composition ratios relative to Ni that are generally consistent with those of chondritic values. This general consistency with a chondritic initial composition for the refractory siderophiles is expected for a bulk core composition and thus also supports the assumption in this model that the lowest As IIIAB irons represent the first solids to form from a fully molten core. In contrast, most elements with lower 50% condensation temperatures are depleted relative to Ni in the IIIAB bulk core composition in comparison to their chondritic ratios. One notable exception is Au, where the modeled IIIAB bulk core composition relative to Ni is similar to its chondritic value. The most recent evaluation by Wood et al. (2019) gives the 50% condensation temperature of Au as 967 K, while earlier studies reported higher values of 1060 K (Lodders 2003) or 1225 K (Wasson 1985), which would make the lack of a Au depletion less striking in Fig. 6 if plotted at these previous higher 50% condensation temperature values.

Depletions are expected in Fig. 6 for any elements that do not partition nearly entirely into the core. For example, the depletion of Fe in Fig. 6 is consistent with the generally expected behavior that while Fe partitioned into the metallic core, some Fe also remained in the silicate portion of the IIIAB parent body during the core formation process. However, most other elements plotted on Fig. 6 are expected to partition strongly into the core based on their metal‐silicate partitioning behavior (Righter 2003). Overall, the bulk composition of the IIIAB core shown in Fig. 6 is consistent with roughly expected chondritic amounts of refractory siderophile elements but with more volatile siderophile elements being depleted in the IIIAB parent body.

It is worth noting that though S has the lowest condensation temperature plotted in Fig. 6, S does not show the lowest depletion, in particular with Sb and Ge being lower. This implies factors other than or beyond volatile depletion based on an element’s 50% condensation temperature and the separation of metal and silicate during differentiation were also responsible for setting the IIIAB initial bulk core composition. Vapor loss during impacts is one option to produce depletions in volatile elements, as has been suggested for the IVA group that exhibits a large range of cooling rates among its members (Yang et al. 2008). The metallographic cooling rates of IIIAB irons also exhibit a range of values (Yang and Goldstein 2006), which if also due to a large impact event during the evolution of the parent body could have resulted in the depletion of volatile elements.

Norris and Wood (2017) demonstrated that volatile depletions can also arise from silicate melts over a range of oxygen fugacities, resulting in depletion patterns that differ from those controlled by condensation temperatures from a gas phase. Of the elements plotted in Fig. 6, the study of Norris and Wood (2017) included four of these elements and found that the increasing volatility of these four elements went in the order of Ga, Cu, Ge, and Sb at low oxygen fugacities. This relative volatility order does not match the relative depletions shown for these four elements in Fig. 6, with Cu being less depleted than Ga, but it is generally consistent. If volatile depletions were set by melt‐vapor reactions on the parent body during differentiation when the body was molten, the volatility of Au under such conditions might be quite different than the volatility expected from its condensation temperature, perhaps explaining the lack of an Au depletion in Fig. 6. Future studies that determine the volatility loss of Au from silicate melts could test this idea and thus help constrain the origin of volatile depletions seen in iron meteorite groups like the IIIAB.

## Summary

The revised trapped melt model presented in this work provides a consistent explanation for the fractionation trends observed in the IIIAB iron meteorite group. In particular, this model is an advancement over previous models for this group for a few key reasons and associated implications:

1. *Inclusion of the effect of the formation of troilite on the solid metal composition*. The revised trapped melt model builds on the earlier conceptual model of Wasson (1999) that trapped melt was an important process during the solidification of the IIIAB core. However, unlike the previous trapped melt model that modeled IIIAB irons as mixtures between solid and liquid metal, the revised trapped melt model includes the additional step that the trapped liquid metal will solidify to troilite and solid metal. The formation of troilite affects the composition of the solid metal that forms from a trapped melt. Measurements of the metallic phase of iron meteorites avoid non‐metallic inclusions such as troilite, and thus to model those measurements of the metal composition, it is necessary to include the formation of troilite in the model calculations. The revised trapped melt model does this for the first time.
2. *Use of experimentally constrained elemental partitioning values while also including the effects of trapped melt*. Previous trapped melt models allowed the element partitioning behavior to vary as a free parameter of the model to produce the best fits. Consequently, even different applications of the trapped melt model had inconsistent element partitioning behaviors between each other. Previous crystallization models that used experimentally determined partition coefficients were not able to explain the scatter in the IIIAB fractionation trends, as mixing between the modeled solid and liquid curves did not fit the IIIAB irons. Consequently, this revised trapped melt model is the first that uses partition coefficients set by experimental measurements that can also explain the scatter in the IIIAB trends as due to effects from trapped melt.
3. *Simultaneous modeling of the largest number of elements for the IIIAB group*. This revised trapped melt model considered 16 elements as well as S and Fe. In particular, 12 of those elements, those without affinities for schreibersite or troilite, were used to constrain and evaluate the model. Using the revised trapped melt model, a solution with an initial S composition of 9 ± 1 wt% S was found to successfully reproduce the trends of all 12 of these elements for the IIIAB iron meteorite group. This is the most elements successfully included in any crystallization model for the IIIAB group.
4. *Implications from the IIIAB bulk core composition*. In addition to providing confidence in the model approach by fitting many elements simultaneously, this result enables the most complete look at the modeled bulk composition of the IIIAB core, which appears consistent with that expected for chondritic contributions of refractory siderophile elements but shows evidence for depletion of more volatile elements. An initial bulk composition of ~9 wt% S implies that there is a substantial amount of S‐rich material associated with the IIIAB core that is underrepresented in our meteorite collections. Trapping S‐rich melt throughout crystallization of the IIIAB core but then removing that S‐rich material from our samples requires an explanation, and processes such as ferrovolcanism or the preferential removal of such phases during a sample’s journey to Earth have been suggested as possibilities.

While this study is focused on the IIIAB iron meteorite group, the results have implications for investigating other magmatic iron meteorite groups as well. In addition to the IIIAB group, the previous trapped melt model has been applied to the IVA (Wasson and Richardson 2001; Wasson et al. 2006; McCoy et al. 2011), IIAB (Wasson et al. 2007), IID (Wasson and Huber 2006), and IVB (Walker et al. 2008) groups, and these results should be revisited using this revised trapped melt model. Additionally, the IVB and IID groups have been suggested to sample cores formed in the outer solar system and the IIIAB, IVA, and IIAB groups to sample inner solar system cores (Kruijer et al. 2017). Applying the revised trapped melt model to these iron meteorite groups and others can enable a new understanding of how the process of trapped melt varied during the crystallization of irons on different bodies across the early solar system. Understanding the role of trapped melt during core crystallization also provides insight into the mode of crystallization of the core, either by concentric front growth or the growth of large dendrites (Haack and Scott 1992; Chabot and Haack 2006); iron meteorite groups that have chemical signatures of extensive trapped melt throughout the crystallization sequence must have had some way to trap the more buoyant melt, implying a more complicated growth than simple concentric crystallization. The mode of core crystallization has implications for the potential generation of asteroidal dynamos, such as discussed for the magnetic signatures recorded in IVA irons as evidence for a core dynamo (Bryson et al. 2017). Modeling many elements simultaneously for these groups will also enable new insights into their bulk core compositions, in particular for the light element of S that is not obtained by direct measurements of the metallic phases of iron meteorites, which will enable comparisons of the variability of planetary core compositions across the solar system.

Overall, this study outlines a revised approach to model the crystallization of magmatic iron meteorite groups and demonstrates its success on the IIIAB group, the largest magmatic iron meteorite group. Future work that builds on this initial study and applies this revised trapped melt model to additional iron meteorite groups can enable new comparisons and insights into the variability of core compositions and crystallization processes in the early solar system.

## Supporting information


**Fig. S1**. Initial 8 wt% S model, applied to: (A) P, (B) Cr, (C), Co, (D) Ni, (E) Cu, (F) Ga, (G) Ge, (H) Ru, (I) Sb, (J) W, (K) Re, (L) Os, (M), Ir, (N) Pt, and (O) Au vs. As. The two mixing lines are shown at 25% and 61% crystallization.
**Fig. S2**. Initial 9 wt% S model, applied to: (A) P, (B) Cr, (C), Co, (D) Ni, (E) Cu, (F) Ga, (G) Ge, (H) Ru, (I) Sb, (J) W, (K) Re, (L) Os, (M), Ir, (N) Pt, and (O) Au vs. As. The two mixing lines are shown at 28% and 56% crystallization.
**Fig. S3**. Initial 10 wt% S model, applied to: (A) P, (B) Cr, (C), Co, (D) Ni, (E) Cu, (F) Ga, (G) Ge, (H) Ru, (I) Sb, (J) W, (K) Re, (L) Os, (M), Ir, (N) Pt, and (O)Au vs. As. The two mixing lines are shown at 22% and 52% crystallization.

**Table S1**. Additional details about some of the irons in Table 1.

**Table S2**. IIIAB irons analyzed at UCLA but not listed in Table 1.

**Table S3**. P composition of IIIAB irons.

**Table S4**. Cape York Compositions.

**File S1**. Tabular output of model run with 8 wt% S.

**File S2**. Tabular output of model run with 9 wt% S.

**File S3**. Tabular output of model run with 10 wt% S.

## Data Availability

The data that support the findings of this study are available in the tables of this article and in the supplementary material provided with this article.
