# Supplementary material for: A revised trapped melt model for iron meteorites applied to the IIIAB group
Source: Meteorit Planet Sci. 2021 Oct 18;57(2):200–27. doi: 10.1111/maps.13740 (PMC9298042; doi:10.1111/maps.13740)
Supplement: Supplementary file 1 — Fig. S1. Initial 8 wt% S model, applied to: (A) P, (B) Cr, (C), Co, (D) Ni, (E) Cu, (F) Ga, (G) Ge, (H) Ru, (I) Sb, (J) W, (K) Re, (L) Os, (M), Ir, (N) Pt, and (O) Au vs. As. The two mixing lines are shown at 25% and 61% crystallization. Fig. S2. Initial 9 wt% S model, applied to: (A) P, (B) Cr, (C), Co, (D) Ni, (E) Cu, (F) Ga, (G) Ge, (H) Ru, (I) Sb, (J) W, (K) Re, (L) Os, (M), Ir, (N) Pt, and (O) Au vs. As. The two mixing lines are shown at 28% and 56% crystallization. Fig. S3. Initial 10 wt% S model, applied to: (A) P, (B) Cr, (C), Co, (D) Ni, (E) Cu, (F) Ga, (G) Ge, (H) Ru, (I) Sb, (J) W, (K) Re, (L) Os, (M), Ir, (N) Pt, and (O)Au vs. As. The two mixing lines are shown at 22% and 52% crystallization. [file MAPS-57-200-s003.docx]

**Supplementary Material**

**A Revised Trapped Melt Model for Iron Meteorites Applied to the IIIAB Group**

Nancy L. Chabot and Bidong Zhang

Table S1 provides additional details about the analyses presented in Table 1 for certain meteorites. Table S2 lists details of seven IIIAB iron meteorites that have appeared in previous UCLA publications with an explanation for why each is not included in Table 1. Table S3 tabulates previously reported P data for IIIAB irons from Buchwald (1975), Doan and Goldstein (1969), Moore et al. (1969), and Lewis and Moore (1971). Table S4 provides measurements of 15 elements in IIIAB Cape York irons. The mean values of each synonym meteorite of Cape York are calculated from their corresponding subsamples. Description of Cape York samples can be found in Esbensen et al. (1982), Esbensen and Buchwald (1982), and Buchwald (1987).

Figures S1-S3 show best fits to the IIIAB iron meteorite trends using the revised trapped melt model for initial S contents of 8, 9, and 10 wt%. Our preferred best-fit value is 9 wt% S, but a slightly lower S content provides better fits to some elements such as Ga and Ge, while other elements, such as Ir, are better fit at a slightly higher S content. Providing model results for these three different S contents provides a way to assess the uncertainties in the initial IIIAB core composition. These uncertainties are primarily attributed to the limitations of the precision with which we know the partitioning behavior of each element as a function of the evolving liquid metal composition. Model outputs as comma-separated values (csv) files (File S1-S3) are provided for each model with 8, 9, or 10 wt% initial S content.

***
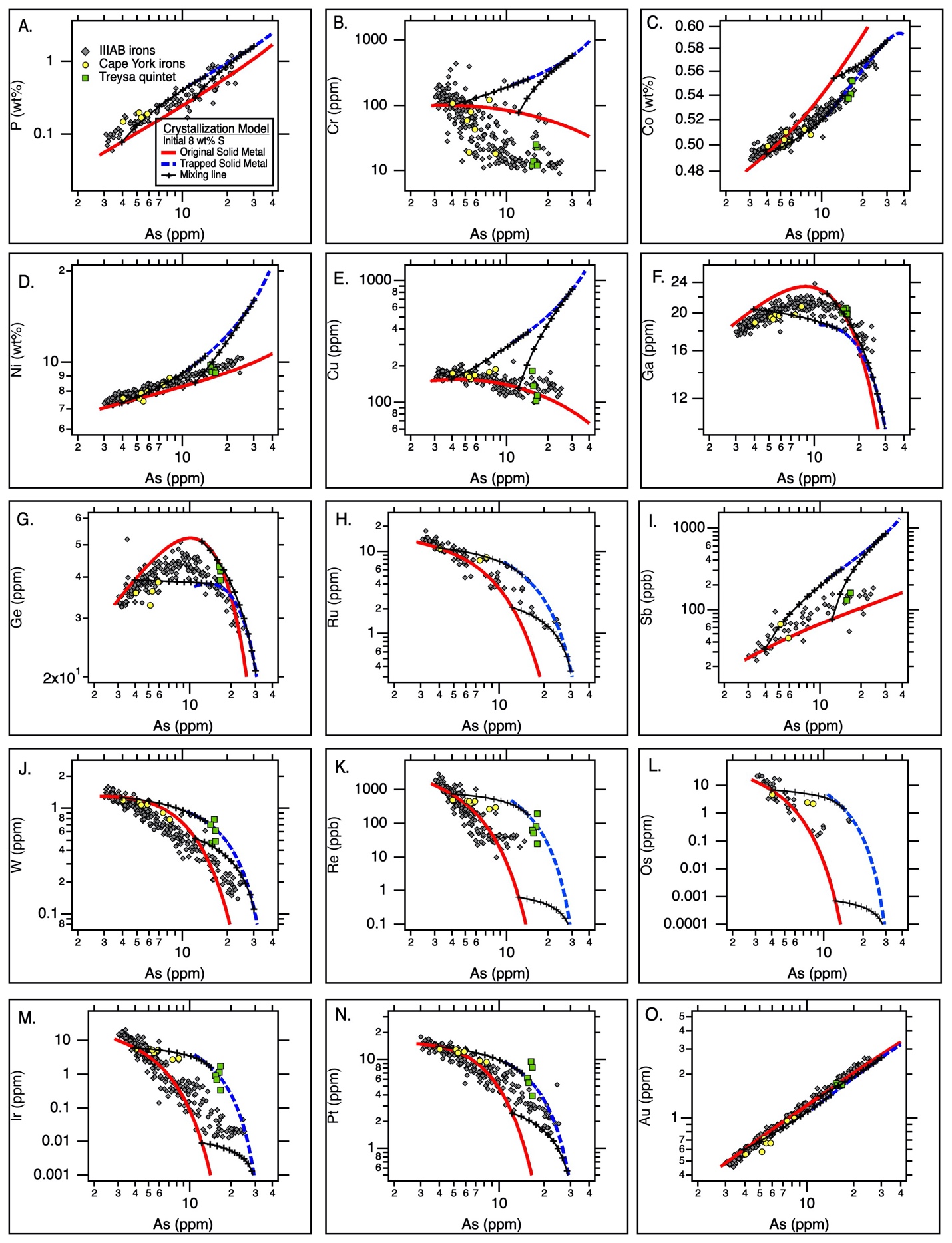
Figure S1.*** *Initial 8 wt% S model, applied to: (****A****) P, (****B****) Cr, (****C****), Co, (****D****) Ni, (****E****) Cu, (****F****) Ga, (****G****) Ge, (****H****) Ru, (****I****) Sb, (****J****) W, (****K****) Re, (****L****) Os, (****M****), Ir, (****N****) Pt, and (****O****) Au vs. As. The two mixing lines are shown at 25% and 61% crystallization.*

*
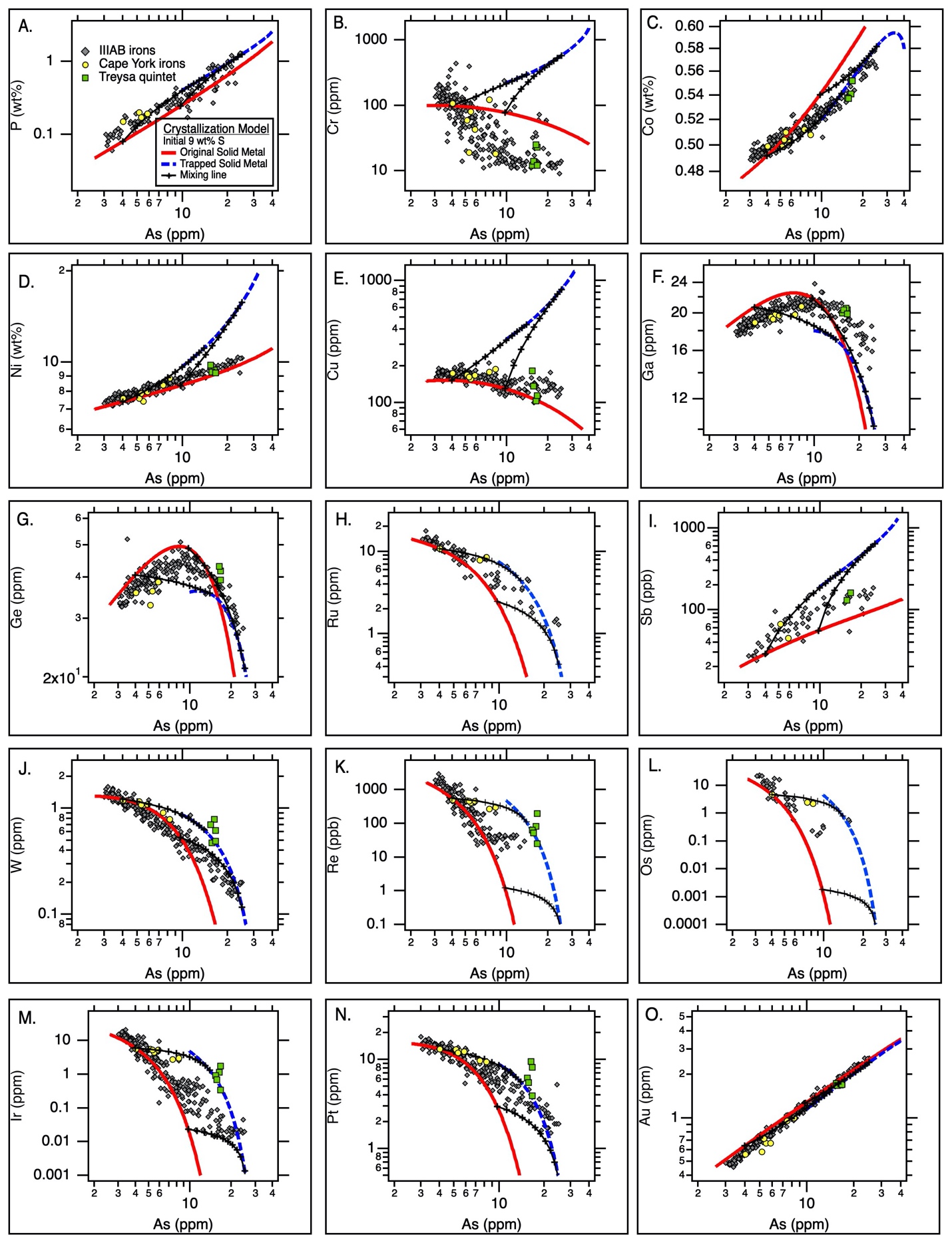
*

***Figure S2.*** *Initial 9 wt% S model, applied to: (****A****) P, (****B****) Cr, (****C****), Co, (****D****) Ni, (****E****) Cu, (****F****) Ga, (****G****) Ge, (****H****) Ru, (****I****) Sb, (****J****) W, (****K****) Re, (****L****) Os, (****M****), Ir, (****N****) Pt, and (****O****) Au vs. As. The two mixing lines are shown at 28% and 56% crystallization.*

*
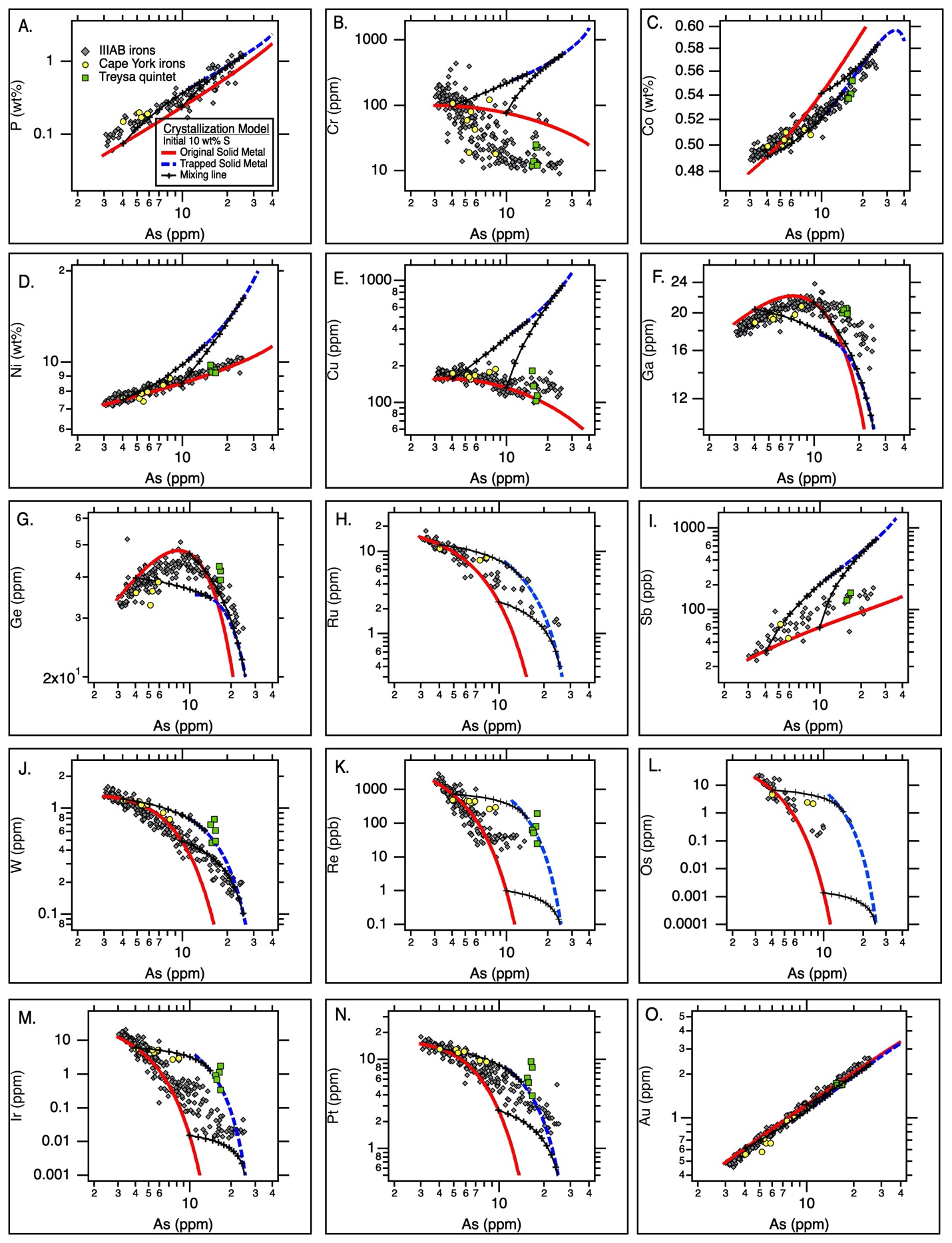
*

***Figure S3.*** *Initial 10 wt% S model, applied to: (****A****) P, (****B****) Cr, (****C****), Co, (****D****) Ni, (****E****) Cu, (****F****) Ga, (****G****) Ge, (****H****) Ru, (****I****) Sb, (****J****) W, (****K****) Re, (****L****) Os, (****M****), Ir, (****N****) Pt, and (****O****)Au vs. As. The two mixing lines are shown at 22% and 52% crystallization.*
